# Microplastic Categories Distinctively Impact Wastewater Bacterial Taxonomic Composition and Antimicrobial Resistance Genes

**DOI:** 10.3390/microorganisms13020260

**Published:** 2025-01-24

**Authors:** Tam Thanh Tran, Kabelo Stephans Stenger, Marte Strømmen, Cornelius Carlos Bezuidenhout, Odd-Gunnar Wikmark

**Affiliations:** 1 Norwegian Research Centre AS (NORCE), Nygårdstangen, 5838 Bergen, Norway; marte-strommen@live.no (M.S.); ogwi@norceresearch.no (O.-G.W.); 2 Unit for Environmental Sciences and Management–Microbiology, North-West University, Potchefstroom 2520, South Africa; kabelostenger@gmail.com (K.S.S.); carlos.bezuidenhout@nwu.ac.za (C.C.B.)

**Keywords:** wastewater treatment plants, microplastics, antimicrobial resistance (AMR), bacterial community, aging treatment

## Abstract

Wastewater treatment plants (WWTPs) may serve as hotspots for pathogens and promote antimicrobial resistance (AMR). Plastic debris in wastewater could further contribute to AMR dissemination. The aim of this study was to investigate the impact of various microplastic types on bacterial communities and AMR gene abundance in wastewater that were obtained from two WWTPs, one in Tromsø, Norway, and the other one in Potchefstroom, South Africa. The microcosm experiments were designed as follows: Five manufactured microplastic pellet types were used for testing, and two rock aggregate types were used as controls. In addition, each material type was subjected to artificial aging treatments using either ultra-violet light or hydrogen peroxide. Each material was incubated in flasks containing inlet/outlet wastewater obtained from these two WWTPs. Nucleic acids were extracted after a one-week incubation period. The detection of the *bla*_FOX_ *and bla*_MOX_ genes was performed using quantitative PCR. Extracted DNA was sequenced using a MinION device. Non-metric multi-dimensional scaling plot on full-length 16S sequencing data at the species level showed that samples were clustered into distinct material groups, which were in line with the ANOSIM test. The Indicator Species Analysis showed a strong association between many *Acinetobacter* species with the plastic group than the rock group. Aging treatment using hydrogen peroxide showed some effects on microbial composition in the outlet wastewater. The abundance of *bla*_FOX_ and *bla*_MOX_ genes in the Norwegian wastewater outlet were generally lower compared to those in the inlet, though the results were contrary in South African wastewater samples. The relative abundance of AMR genes seemed to be increased on several plastic types (PET, PE, and PLA) but decreased on PVC-A. WWTP treatments in this study did not effectively reduce the abundance of AMR genes. An in-depth understanding the role of specific microplastic type on bacterial communities and AMR profiles is, therefore, needed to combat AMR threat.

## 1. Introduction

Antimicrobial resistance (AMR) is one of top 10 global threats according to the World Health Organization WHO [1]. Deaths attributable to AMR continue to rise alarmingly towards the number of 10 million deaths annually which was predicted in the 2016 report [2]. In 2019, the death toll attributed to AMR was reported to be about 4.95 million, which exceeded other causes of death such HIV/AIDS, breast cancer, and malaria [3]. The Quadripartite organizations developed the ‘One Health Priority Research Agenda’ to mitigate this threat and support national action plan implementation and the achievement of the Sustainable Development Goals for 2030 [4]. One Health is a term used to describe the health-related interconnection between humans, animals, and their living environments [5,6]. The One Health approach has been widely adopted by many organizations/government bodies such as the EU, WHO, and United Nations [4,7,8].

Human and animal effluents and solid wastes from humans (including healthcare facilities and community settings) and from agri-food systems (including consumers) have been known as sources of pathogens and AMR genes [9]. Waste streams excreted from anthropogenic activities largely contributes to the dissemination of pathogens and AMR genes into the surrounding environments if they do not get treated properly, which mainly takes place at wastewater treatment plants (WWTPs) [9,10]. However, not all treatment methods result in the safe discharge of effluents into the environment.

The *ampC* genes are chromosomally encoded on the genomes of various members of Enterobacteriaceae and can also be found as plasmid-mediated *ampC* genes belonging to class C β-lactamases, which include six families: CIT, ACC, EBC, DHA, MOX, and FOX. AmpC β-lactamases are clinically important cephalosporinases that confer resistance to cephalothin, cefazolin, cefoxitin, and penicillins. These antibiotics are among the most important therapeutic options used for the treatment of a plethora of infectious diseases mediated by *Enterobacter* spp., *Mycobacterium* spp., *Acinetobacter* spp., *Salmonella* spp., etc. [11,12].

WWTPs are collection points for microplastics present in domestic wastewater, industrial wastewater, and rainwater runoff. Microplastics in wastewater can be detected in sizes ranging from 20 to 5000 µM, depending on the sieve size, and in various shapes such as foils, fibres, spheres, pellet, fragment, foam, and film [13]. Therefore, microplastics present in WWTP could pose a risk in aquatic environments, owing to their ability to act as vectors of other environmental contaminants (antibiotic, ARGs, and pathogens) present in wastewater. For instance, microplastics can act as carriers for pathogens derived from wastewater such as *Arcobacter* spp., *Aeromonas* spp., and *Vibrio* spp. [14] while enhancing the horizontal gene transfer of ARGs in wastewater [15]. Moreover, microplastics can alter the distribution and removal of microbial communities and ARGs in treated wastewater [16,17,18]. In view of these factors, microplastics discharged in treated effluent serves as an important pathway for propagating antibiotic resistance in both terrestrial and aquatic food chains, posing a health threat to both human and livestock [14].

There are different treatment processes that can be used either alone or in combination to treat wastewater such as membrane filtration, coagulation, adsorption, and advanced oxidation processes [9]. Depending on geographical locations, wastewater profiles are drastically different; hence, the same treatment method could lead to various outcomes in wastewater discharge. The selection of appropriate treatment processes for a specific location is critical in eliminating/reducing the disposal of pollutants into the environment as well as in AMR monitoring. WWTP processes are reported to remove over 90% of microplastics in the final effluent [13,19,20], with the smallest particles showing the lowest removal efficiency [19]. For instance, WWTPs in China [21,22], the UK [20], and Turkey [13] reported daily microplastic discharges of approximately 2.87 × 10^8^–6.5 × 10^8^, 1.2 × 10^7^, and 1.8 × 10^7^ microplastic particles, respectively. These WWTPs effectively eliminated microplastics with removal efficiencies of 96–97%, 96%, and 97%, respectively. Despite the high removal efficiency, large amounts of microplastics escape from WWTPs into aquatic environments, as such WWTPs remain a major emission source of microplastics [13].

Microbial colonization on plastic debris was documented for the first time more than fifty years ago [23]. However, with the ever-increasing human use of plastics, more and more plastic debris have been polluting natural habitats and other environments. There are several studies on the effect of different types of microplastics on bacterial communities [24,25,26,27,28]. For instance, different microplastic types impacted differently on the growth of marine bacterial biofilm and bacterial composition in anaerobic laboratory microcosms [25]. Polystyrene was shown to develop a distinct bacterial community compared to those of polyethylene and propylene [24]. A more recent study also reported that biofilm formation and bacterial diversity were distinct among these three microplastics (polyethylene, polypropylene, and polystyrene) [27]. However, these studies mainly focused on the impact of microplastics on marine-originated bacterial community. The role of microplastics in disseminating AMR genes is also understudied.

Materials are likely weathered through oxidation/photodegradation processes when exposed to chemicals or sunlight in nature [29,30,31]. The degradation mechanism when exposed to sunlight, also termed as ‘photodegradation’, has been well studied and is caused by the absorption of photons from several wavelengths found in sunlight, including infrared radiation, visible light, and ultra-violet light [29,30]. This mechanism can occur in the absence of oxygen (chain breaking or cross-linking) or the presence of oxygen (photooxidation). Material exposed to hydrogen peroxide, a strong oxidizing agent, tended to be degraded or have structural changes [32]. Aged microplastics were shown to alter microbial community structure in soils, rivers, and costal areas [33,34,35]. However, more studies are still needed to understand the effect of aged materials on microbial community, especially from wastewater.

The main purpose of this study was to investigate the impact of various microplastic types on bacterial community and AMR gene abundance in two WWTPs, one in Norway and one in South Africa. This study also further investigated the effect of two artificial plastic aging treatments on these bacterial communities.

## 2. Material and Method

### 2.1. Experimental Set-Up

Inlet and outlet wastewater samples were obtained from a local wastewater treatment facility in Tromsø, Norway, and Potchefstroom, South Africa. A volume of 100 mL wastewater was added into sterile flasks containing each material type (plastics or rocks), with an amount equivalent to 10 mL volume. A sterile flask containing only wastewater was also included at the same time. These flasks were incubated at room temperature (around 20 °C) for seven days in a static condition.

Five plastic types in pellet forms were used in this study: high-density polyethylene (HDPE), polyvinyl chloride (PVC.A), polyethylene terephthalate (PET), polylactic acid (PLA), and polyethylene (PE) (purchased from Plastics SA, Midrand, South Africa). They have round, sphere, and almost uniform shape with sizes ranging between 2 and 5 mm. Two types of rocks were purchased from a local store in Tromsø, Norway (Pets Tromsø Dyrebutikk): Black rock (Merkur 3–5 mm, 3 Ltr, AT20503), White rock (Sirius—Hvid AT22410 3–5 mm). Rocks have a bit more irregular shapes: Black rock has a slightly bigger size around 5–10 mm, and White rock has a size between 3 and 5 mm.

Before being used, control materials were sterilized by soaking in bleach for 30 min. Materials were aged by being exposed under UV light with a wavelength of 254 nm or being soaked in hydrogen peroxide 33% (Catalog no. 23613.297, VWR Chemicals BDH^®^, Radnor, PA, USA) in 1 or 24 h (overnight). All material were then rinsed with antibact^®^ solution (ethanol 75% base solution—KiiltoClean AS, Asker, Norway) for 5 min and left to air-dry in the biosafety cabinet. A volume of 100 mL wastewater was also filtered using 0.22 µm MCE Membrane Filter (Catalog no. GSWP04700, Millipore^®^, Burlington, MA, USA). Each control/aged material set-up was performed in triplicate. In some experiments, aging treatment using UV was excluded due to large variation among sample replicates.

### 2.2. DNA Extraction and Quantification

Water filter/plastics/rocks were collected and washed once with 10 mL of phosphate-buffered saline (PBS) + tween 0.1% buffer. Then, 10 mL of the same buffer was added into plastic pellets and vortexed to a low to high speed for 1 min to resuspend bacterial biofilm from plastics/rocks/filter. Cells were harvested by being centrifuged at 12,000× *g*, 1 min (repeating the steps until finished), and the liquid was discarded. The cell pellet was resuspended in 300 µL PBS + tween 0.1% buffer; then, 97 µL lysozyme solution (10 mg/mL) and 1 µL RNAse A were added and incubated at 37 °C for 30 min. Subsequently, DNA was extracted using the phenol-chloroform protocol [36]. DNA pellet was finally dissolved in 100 µL of Tris buffer.

DNA was quantified using both NanoDrop 2000c spectrophotometer (ThermoFisher Scientific, Oslo, Norway) and Qubit 4 Fluorometer (ThermoFisher Scientific, Oslo, Norway) according to the manufacturer’s protocol.

### 2.3. Quantitative PCR Analysis to Detect and Quantify AmpC β-Lactamase Gene Groups

A total of 48 samples incubated with inlet and outlet wastewater in each country were used for this qPCR analysis. DNA samples from Norway were shipped to South Africa. The analysis was performed in a lab in North-West University, Potchefstroom, South Africa.

The quantification of AmpC β-lactamase gene groups (*bla*_FOX_ and *bla*_MOX_/*bla*_CMY_) was performed using QuantStudioTM 3 System v1.4.3 (Applied Biosystems, Thermo Fisher Scientific, Waltham, MA, USA). The reaction mixture consisted of 1× TaqManTM Fast Advanced MasterMix (Thermo Fisher Scientific, USA), 1× TaqMan assay, DNA template, and nuclease-free water to a total reaction volume of 10 µL. Thermal cycling conditions consisted of a holding stage with step 1 at 50 °C for 2 min and step 2 at 95 °C for 10 min. The PCR stage consisted of 40 cycles with step 1 at 95 °C for 15 secs followed by a final step 2 at 60 °C for 1 min.

The following FAM fluorescent dyes were used for quantification: Pa04646126 (*bla*_FOX_) and Pa04646156_s1 (*bla*_MOX_/*bla*_CMY_). Standard curves were generated using positive control samples for each target gene containing known copies using a ten-fold dilutions series in triplicates ranging between 20 000 and 2 copies. Amplification efficiencies of standard curves between 90% and 110% (E = 10 − 1/slope − 1) and R2 > 0.97 were deemed as reliable [12]. DNA was extracted from Norwegian samples in Norway, and then cold shipped to a lab in Potchefstroom, South Africa, to perform this qPCR along with South African samples.

### 2.4. MinION Sequencing Protocol

A total of 76 Norwegian and 43 South African samples were used for 16S amplicon sequencing, and 12 Norwegian samples were used for metagenomic sequencing. Sequencing was performed locally in each country using the same protocol.

DNA sequencing was performed on either a flongle FLO-FLG001 or MinION FLO-MIN106 flow cell (R9.4.1) using long-read Oxford Nanopore MinION platform (Oxford Nanopore Technologies Ltd., Oxford, UK). DNA libraries were prepared and loaded on flow cells following the default manufacturer protocol for 16S barcoding (the SQK-RAB204 or SQK-16S024 kit) or rapid barcoding (the SQK-RBK004 kit) for metagenomic sequencing. MinION Mk1C installed with software version 23.04.5 (Oxford Nanopore Technologies) was used to perform sequencing. Each multiplexed run for 16S amplicon sequencing produced between 24 and 381,280 reads per sample with the mean read length of around 1.4 kb. The multiplexed run for metagenomic sequencing produced between 9311 and 318,137 reads with a mean read length of 3.65 kb. Guppy v. 6.0.6 and v. 6.5.7 (Oxford Nanopore Technologies) were used to perform demultiplexing, base-calling, and quality filtering of the raw reads for 16S and metagenomic sequencing, respectively.

### 2.5. Bioinformatic Analyses

Full-length 16S fastq reads were further analyzed using Fastq 16S v2022.01.07 on EPI2ME Desktop Agent v3.5.5 with the following default settings: min qscore of 7, BLAST e-value filter of 0.01, minimum coverage of 30%, and min identity of 77%. The reports were then exported and saved as csv files so that further analyses could be performed using R or python programming.

Metagenomic fastq reads were analyzed directly using Fastq Antimicrobial Resistance v2023.04.26 EPI2ME Desktop Agent v. 3.5.5 with default settings. The results were exported in tsv files in the epi2me output folder, and further analysis was performed using python programming. Metagenomic fastq reads were further assembled using the Flye v. 2.9.2 tool [37]. The assemblies were used as the input to MOB-suite v. 3.1.0 to characterize the plasmid content, and StarAMR v. 0.9.1 to detect antibiotic resistance genes based on the resfinder resistance gene database [38,39,40].

All sequencing data are deposited in the National Center for Biotechnology Information (NCBI) Sequence Read Archive under BioProject ID PRJNA1061070.

### 2.6. Visualization and Statistical Analyses

Statistical analysis was performed using either JASP software (Version 0.17.1) or R version 4.2.2 (31 October 2022 ucrt) on RStudio 2023.06.0 Build 421 (© 2009–2023 Posit Software, PBC). If assumptions for normality (Shapiro–Wilk) and the homogeneity of variance (Levene’s Test) were met, the *t*-test or one-way ANOVA was used to determine whether the difference was statistically significant (*p* < 0.05). If the above assumptions were not met, the Mann–Whitney or Kruskal–Wallis rank sum test was used instead. To determine whether the difference observed in the abundance of ARGs on microplastics substrates and natural substrates was statistically significant (*p* < 0.05), one-way ANOVA and Tukey’s HSD test were used.

For visualization, two R packages were used in this study, ‘vegan’ and ‘ggplot2’, for non-metric multi-dimensional scaling (NMDS) and bar plots. For statistical analyses, the non-parametric ANOSIM test with the number of permutations of 999 and the Bray–Curtis dissimilarity indices in the package ‘vegan’ was performed to determine if there is a statistical difference between the microbial communities of two or more groups of samples. The Indicator Species Analysis in the ‘indicspecies’ package was used to identify microbial species that are found more often in one treatment group compared to another.

## 3. Results

### 3.1. Impact of Material and Aging Treatment on DNA Extraction

**Figure 1:**
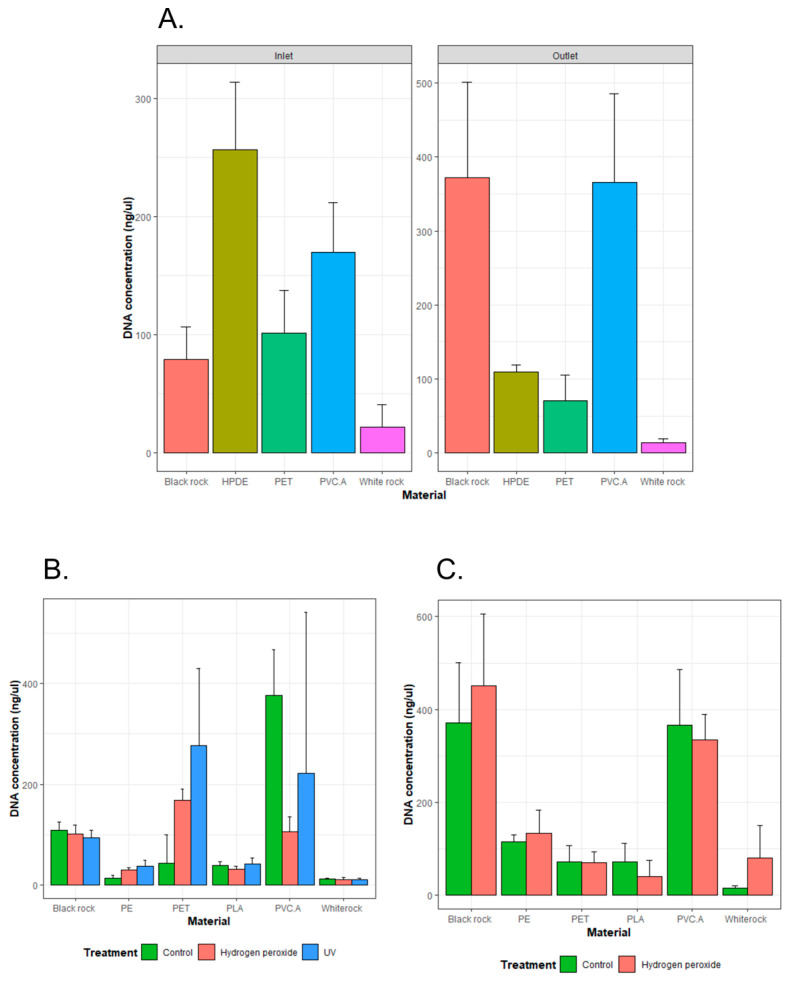
DNA obtained from different pristine materials: Black rock, HDPE, PET, PVC.A, and White rock incubated in inlet and outlet wastewater (**A**), different pristine or aged materials (using hydrogen peroxide or UV for a 24 h period) incubated in wastewater—(**B**): inlet; (**C**): outlet. Inlet and outlet wastewater were obtained from a Norwegian WWTP.

DNA was extracted most from HDPE and PVC.A materials in the inlet wastewater set-up, while Black rock and PVC.A produced more DNA in the outlet wastewater set-up (Figure 1A). White rock produced the least DNA in both inlet and outlet wastewater set-ups. Given that assumptions were met, one-way ANOVA showed statistically significant *p*-values when comparing DNA concentration obtained from different materials in both set-ups (Inlet: *p* < 0.0001; Outlet: *p* < 0.0001). The Tukey’s HSD post hoc test for pairwise comparisons showed these following group pairs significantly different in both set-ups (*p* < 0.05): HDPE—Black rock, PVC.A—PET, White rock—PVC.A.

Aging treatment and its duration posed different effects on extracted DNA depending on the material. For the inlet wastewater set-up, DNA concentrations appeared to increase significantly in aged PE (*p* = 0.01 using hydrogen peroxide and *p* = 0.02 using UV) and PET (*p* = 0.012 using hydrogen peroxide and *p* = 0.035 using UV) (Figure 1B). However, DNA concentration decreased in aged PVC.A after 24 h treatment with hydrogen peroxide (*p* = 0.004). The shortening treatment period significantly increased DNA concentration extracted from aged PVC.A groups compared to control group (*p* = 0.02 using hydrogen peroxide and *p* = 0.026 using UV). Kruskal–Wallis’s test on all these samples showed a significant *p*-value if the grouping was based on the material (*p* < 0.0001) and insignificant *p*-value if the grouping was based on the aging method (*p* > 0.05). For the outlet wastewater set-up, there was no significant difference in DNA concentrations between the control and aged groups for each material (Figure 1C). In addition, Kruskal–Wallis’s test also showed a significant difference when samples were grouped into different material types (*p* < 0.0001), and insignificant difference when samples were grouped into non-aged/aged groups.

### 3.2. Microbial Genera from Inlet and Outlet Wastewater and Their Colonization on Plastics or Rocks

**Figure 2:**
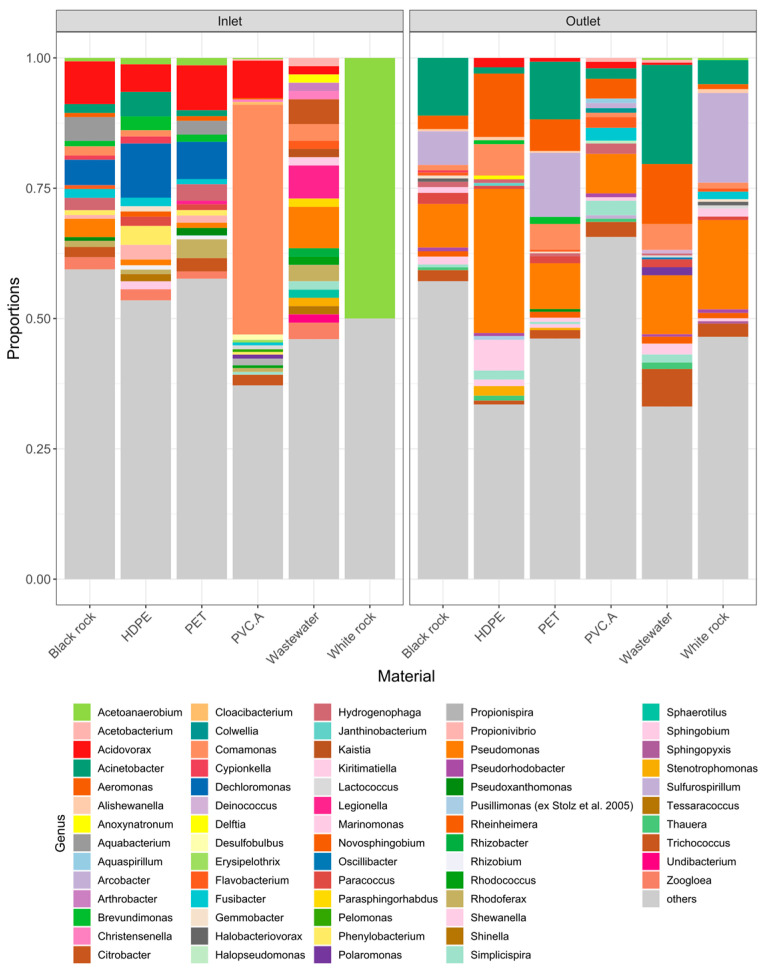
Top 20 bacterial genera detected in each testing material from Norwegian wastewater set-up.

*Acidovorax* and *Dechloromonas* were the most two prevalent genera present in three testing materials incubated with Norwegian wastewater inlet (Figure 2): Black rock (8.2 and 4.9%, respectively), HDPE (10.4 and 5.3%, respectively), and PET (8.6 and 7.1%, respectively). *Comamonas* and *Acidovorax* were abundantly present in PVC.A (44 and 7.1%, respectively). Wastewater inlet seemed to have an even distribution among detected genera. DNA obtained from incubation using white rock in inlet wastewater was very little, resulting in very poor sequencing data.

*Acinetobacter*, *Pseudomonas*, and *Arcobacter* were the most prevalent genera present in three testing materials incubated with Norwegian wastewater outlet (Figure 2): Black rock (11.1, 8.3, and 6.4%, respectively), White rock (4.6, 17.1, and 17.2%, respectively), PET (11.1, 8.8, and 12.3%, respectively). On the other hand, *Pseudomonas* and *Aeromonas* were found more on HDPE (27.6 and 12.2%, respectively) and PVC.A (7.5 and 3.8%, respectively). The top three genera found in wastewater outlet were *Acinetobacter* 19%, *Aeromonas* 11.5%, and *Pseudomonas* 11.3%.

**Figure 3:**
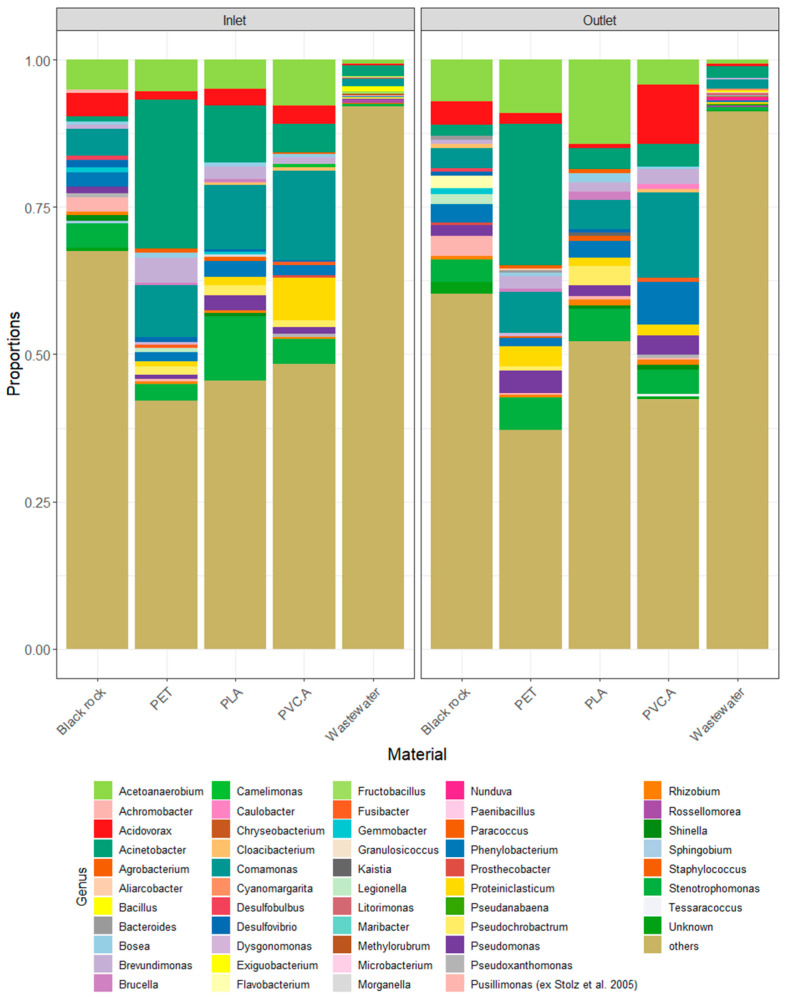
Top 20 bacterial genera detected in each testing material from South African wastewater set-up.

*Comamonas* is among the top three prevalent genera present in four testing material incubated with South African wastewater inlet (Figure 3): Black rock 4.6%, PET 8.9%, PLA 10.8%, and PVC.A 15.2%. Other prevalent genera found in these materials are: *Acetoanaerobium* (Black rock 5.1%, PET 5.4%, PVC.A 7.8%), *Stenotrophomonas* (Black rock 4%, PLA 10.9%), *Acinetobacter* (PET 25.3%, PLA 9.6%), and *Proteiniclasticum* (PVC.A 15.2%). The top three genera found in the wastewater inlet were *Acinetobacter* 1.8%, *Comamonas* 1.2%, and *Exiguobacterium* 0.9%.

The following genera are among the top three prevalent genera present in four testing materials incubated with the South African wastewater outlet (Figure 3): *Comamonas* (PET 7%, PLA 5%, PVC.A 14.5%), *Acetoanaerobium* (Black rock 7.1%, PET 9.1%, PLA 14.3%), *Stenotrophomonas* (Black rock 3.7%, PLA 5%), *Acinetobacter* (PET 24%), *Phenylobacterium* (PVC.A 7.2%), and *Acidovorax* (Black rock 3.9%, PVC.A 10.1%). The top three genera found in the wastewater outlet were *Acinetobacter* 2%, *Comamonas* 1.7%, and *Acetoanaerobium* 0.7%.

### 3.3. Impact of Material Type on Bacterial Colonization at Species Level

**Figure 4:**
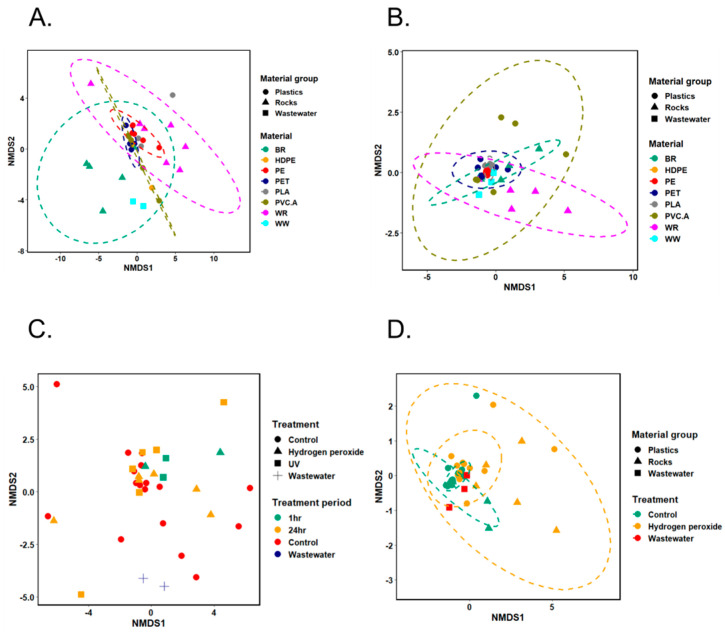
Non-metric multi-dimensional scaling (NMDS) analysis on bacterial community using full-length 16S rRNA gene dataset in microcosm experiment set-up by incubating of microplastics in Norwegian wastewater. (**A**,**B**) Seven different materials (BR: Black rock; WR: White rock; HDPE; PE; PET; PLA; PVC.A) and wastewater (WW)—(**A**): inlet; (**B**): outlet. (**C**) Three treatment groups (control, hydrogen peroxide, and UV) with either 1 h or 24 h periods and inlet WW. (**D**) Two treatment groups (control, hydrogen peroxide—24 h) and outlet WW.

**Figure 5:**
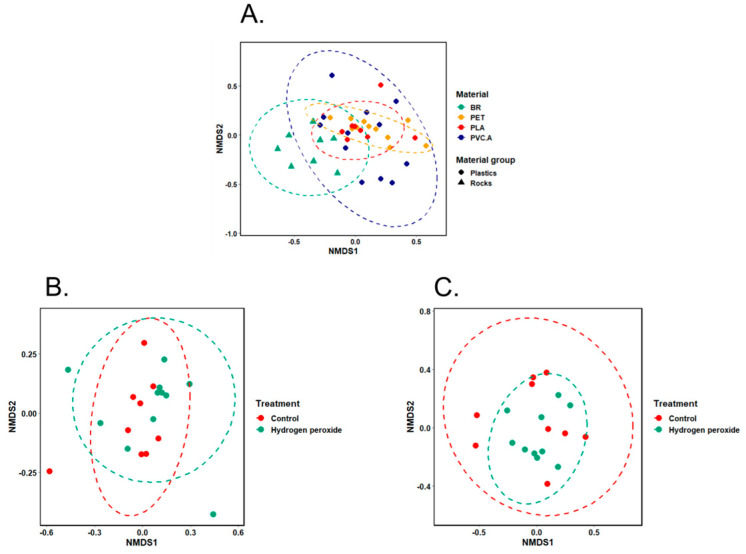
Non-metric multi-dimensional scaling (NMDS) analysis on bacterial community using full-length 16S rRNA gene dataset in microcosm experiment set-up by incubating of microplastics in South African wastewater. (**A**) Four different materials (BR: Black rock; PET; PLA; PVC.A), (**B**) two treatment groups (control and hydrogen peroxide—24 h) in inlet wastewater, and (**C**) two treatment groups (control and hydrogen peroxide—24 h) in outlet wastewater.

The NMDS plots showed that samples were clustered well based on testing materials using bacterial abundance data as input (Figure 4A,B and Figure 5A). This observation was consistent in both inlet and outlet wastewater set-ups and for both Norwegian and South African samples.

For the Norwegian inlet wastewater set-up (Figure 4A), species-level bacterial composition was significantly different among three categories, plastics, rocks, and wastewater (*p* = 0.0005, statistic R = 0.289, the ANOSIM test using Bray–Curtis’s dissimilarity matrices), and among eight specific materials (*p* = 0.0008, statistic R = 0.2501, the ANOSIM test using Bray–Curtis’s dissimilarity matrices). When performing Indicator Species Analysis on the three groups (plastics, rocks, and wastewater), none of species were shown to be strongly associated with any of these groups. However, performing this analysis on specific materials showed a list of species that are likely to be associated with each material and their combined groups (Text S1). *Chryseobacterium treverense* showed some significant association with two plastic types (PET and PE).

For the Norwegian outlet wastewater set-up (Figure 4B), species-level bacterial composition was significantly different among three categories, plastics, rocks, and wastewater (*p* = 0.0087, statistic R = 0.2662, the ANOSIM test using Bray–Curtis’s dissimilarity matrices), and among eight specific materials (*p* = 0.0001, statistic R = 0.2452, the ANOSIM test using Bray–Curtis’s dissimilarity matrices). In this set-up, a list of species that had a strong association with plastic group compared to rock or wastewater groups were able to be identified using Indicator Species Analysis (Text S2). Among those are nine *Pseudomonas*, four *Stenotrophomonas*, three Flavobacterium, and three Sphingobium species. In addition, performing Indicator Species Analysis on these data also listed a list of indicator species for each specific material and their combined groups (Text S3).

For the South African wastewater set-up (Figure 5A), species-level bacterial composition was not significantly different between plastic and rock groups but significantly different among four specific materials (*p* = 0.0001, statistic R = 0.3268, the ANOSIM test using Bray–Curtis’s dissimilarity matrices. When performing Indicator Species Analysis on two groups, plastics and rocks, 97 species were shown to be strongly associated with plastics, and 368 species were strongly associated with rocks (only Black rock was used in this case) (Text S4). Notably, among those species, 38 *Acinetobacter* species showed significant association with the plastic group, and no *Acinetobacter* species showed significant association with the rock group. Performing Indicator Species Analysis on specific materials showed a list of species that are likely to be associated with each material and their combined groups (Text S5). Many *Acinetobacter* species (46 out of 86 species in total) showed a significant association in the PET and PLA combined group.

### 3.4. Impact of Hydrogen Peroxide and UV Used in Aging Treatment on Bacterial Colonization at Species Level

For the Norwegian inlet wastewater experiment set-up, samples from both treatment groups (wastewater, control, UV, and hydrogen peroxide) and treatment period groups (wastewater, control, 1 h and 24 h period) were scattered on NMDS plot (Figure 4C). There was also no significant difference between the microbial communities based on the type of treatment (ANOSIM statistic R: −0.04421 and *p*-value: 0.6942) or the period of treatment (ANOSIM statistic R: −0.04187 and *p*-value: 0.6824).

However, for the Norwegian outlet wastewater experiment set-up, NMDS plot showed some clustering among different treatment groups (wastewater, control, and hydrogen peroxide) (Figure 4D). The ANOSIM test also showed a significant difference in microbial community among these groups (ANOSIM statistic R: 0.1431 and *p*-value: 0.0135). In the outlet set-up, only hydrogen peroxide was used to treat materials for 24 h.

Similar results were also observed for South African inlet and outlet wastewater set-ups (Figure 5B,C). There was no significant difference between the aged and non-aged groups in the inlet set-up (ANOSIM statistic R: 0.0101 and *p*-value: 0.3559), but there was a significant difference in the outlet set-up (ANOSIM statistic R: 0.1868 and *p*-value: 0.0251). Materials were aged by being immersed in hydrogen peroxide for 24 h.

### 3.5. Detection of AMR Genes and Mobile Genetic Sequences in Norwegian Inlet and Outlet Wastewater Using Metagenomic Approach

**Figure 6:**
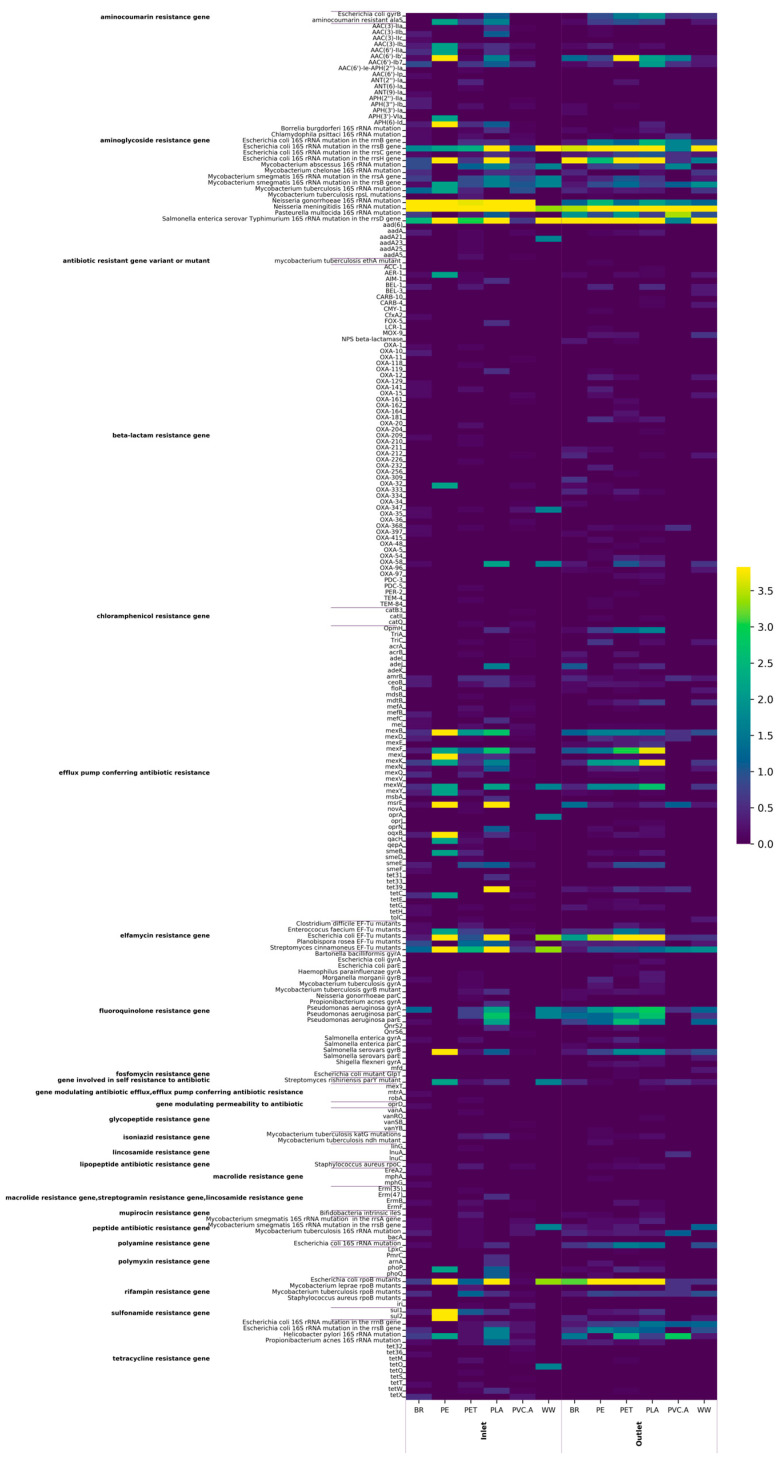
Heatmap diagram showing the relative abundance of AMR genes detected in both inlet and outlet wastewater set-ups. The relative abundance was the proportion of the number of reads for each gene over the number of bacterial reads in each sample. The purple line in the Y-axis grouped genes according to their functions.

Many AMR genes were detected in both inlet and outlet wastewater set-ups using Epi2me pipeline on raw reads (Figure 6). The heatmap showed that certain AMR genes detected on several microplastics (PE, PET, and PLA) abundantly increased in both inlet and outlet set-ups compared to those detected in wastewater or black rock samples. Those genes were found to belong to the following functional groups: aminoglycoside resistance, efflux pump, elfamycin resistance, fluoroquinolone resistance, and rifampicin resistance. To a lesser extent, the proliferation of AMR genes was also observed in beta-lactam resistance, sulfonamide resistance, and tetracycline resistance. However, this was not the case for PVC.A, on which AMR genes were detected less than those on black rock and wastewater samples. The relative abundances of total AMR genes for these samples were also in agreement with the above heatmap results in Figure 6.

The detection of AMR genes on assemblies also showed several acquired resistance genes (Table S1). Notably, these AMR genes were found only from plastic group (PLA, PET, PE). Many insertion sequences were detected on chromosomal assemblies from both inlet and outlet wastewater set-ups (Table S2). In the inlet wastewater, Tn3, IS6, and IS3 were the ones detected on PLA and PET groups. Meanwhile, in the outlet wastewater, a more diverse variety of insertion sequences were detected, especially in the PLA, PET, and PE groups. Only two insertion sequences were detected on plasmid contigs from the PET-outlet group (Table S3).

### 3.6. Quantification of bla_FOX_ and bla_MOX_ Genes in Inlet and Outlet Wastewater

**Figure 7:**
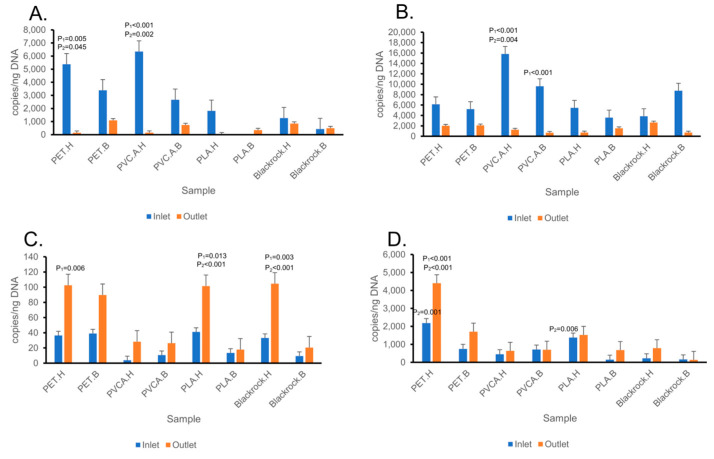
Gene copies per ng DNA of *bla*_FOX_ genes (**A**,**C**) and *bla*_MOX_ genes (**B**,**D**) inlet and outlet wastewater from Tromsø, Norway (**A**,**B**), and South Africa (**C**,**D**). Treatment: H—hydrogen peroxide (aged); B—bleach (control). Significant: P_1_—between groups (inlet–outlet); P_2_—between treatment (aged–control).

In the Norwegian samples, the *bla*_FOX_ and *bla*_MOX_ genes were generally found decreased in the outlet compared to those in the inlet wastewater set-up (Figure 7A,B). When comparing copy numbers of the *bla*_FOX_ gene in the outlet with those in the inlet wastewater set-up, significant decreases were found on aged PET (*p* = 0.005) and aged PVC.A (*p* < 0.001). When comparing copy numbers of the *bla*_MOX_ gene in the outlet wastewater with those in the inlet wastewater set-up, significant decreases were found on non-aged PVC.A (*p* < 0.001) and aged PVC.A (*p* < 0.001). When comparing between aged and non-aged materials, the inlet set-up showed that the *bla*_FOX_ gene copy numbers on aged-PET (*p* = 0.045) and PVC.A (*p* = 0.02) were significantly higher. Similarly, the *bla*_MOX_ gene copy number on aged PVC.A (*p* = 0.04) was also significantly higher.

Meanwhile, in the South African samples, the *bla*_FOX_ and *bla*_MOX_ genes were generally found increased in the outlet compared to those in the inlet wastewater set-up (Figure 7C,D). When comparing copy numbers of the *bla*_FOX_ genes in the outlet wastewater with those in the inlet wastewater set-up, significant increases were found in aged PET (*p* = 0.006), aged PLA (*p* = 0.013), and aged Blackrock (*p* = 0.003). When comparing copy numbers of the *bla*_MOX_ gene in the outlet wastewater with those in the inlet wastewater set-up, a significant increase was found in aged PET (*p* < 0.001). When comparing between aged and non-aged materials, the *bla*_FOX_ gene copy numbers in aged-PLA (*p* < 0.001) and aged-Black rock (*p* < 0.001) were significantly higher in the outlet set-up. The *bla*_MOX_ gene copy numbers were also significantly higher in aged PET (*p* = 0.001) and aged PLA (*p* = 0.006) in the inlet set-up, and in aged PET (*p* < 0.001) in the outlet set-up.

## 4. Discussion

The DNA amount extracted from different testing materials were shown to be significantly different among them. A very little amount of DNA was obtained from White rock, which was consistent across replicates between inlet and outlet set-ups. To our surprise, there was a significant difference in the extracted DNA between White rock and Black rock, although they were purchased from the same pet store for the same use purpose as decorators in aquarium tank. We hypothesize that the compositional difference is the main factor leading to different DNA amounts extracted from these two rocks. The difference in total extracted DNA may result from how well the materials can be served as a ‘habitat’ for microorganisms to grow. Information on original countries where these rocks were sourced were given by the production company Akvastabil, Eldorado A/S, Denmark. The White rock was from Sweden, and the Black rock was from Germany. They both were crushed after being excavated without any further treatment at the factory. The White rock, according to the company, also called ‘Dolomitkross’ (Dolomite crushes), is mainly composed of the mineral dolomite, CaMg(CO_3_)_2_ [41]. It was shown that CaMg(CO_3_)_2_ has some antibacterial properties, and this explains poor DNA obtained from this White rock in both inlet and outlet set-ups [42]. There were also significant differences in the extracted DNA among different types of testing microplastics. More surprisingly, the pattern was not consistent between inlet and outlet set-ups. For example, the DNA obtained from Black rock in the inlet set-up was less than that in the outlet set-up, while the opposite result was observed with HDPE. An explanation for this could be the source of bacteria introduced to these materials. If a specific material favored the colonization of certain bacteria present in the inlet wastewater, the overall DNA outcome would then be better in the inlet set-up than the outlet set-up. This will also be further discussed as we investigate the effect of material on bacterial composition. The DNA extracted here is the total environmental DNA which may include free DNA present in the wastewater samples. These free DNA could possibly be adsorbed on testing materials during the incubation [43,44]. It was shown that different plastic types posed different adsorption affinity on environmental DNA [43].

There is mounting evidence that microplastics play an important role in the colonization of biofilm-forming microorganisms [26,45,46,47]. In our study, there was a significant effect of the material on the microbial community. This observation was seen in both the inlet and outlet wastewater set-up and in Norway and South Africa. The effect of the material on the microbial community was most obvious when the samples were grouped in specific types of materials. This result is in line with several previous studies that showed a significant difference in the microbial community colonizing on different plastic types [24,28,48]. However, the results from a previous study suggested otherwise that plastic polymer type did not play a significant role in shaping biofilm communities [26]. Of note, there are a few differences in how to assess the role of plastic types on microbial communities between our study and others: microbial source, testing plastics, sequencing technology, and taxonomic levels used as input data and statistical models. Among those differences, we supposed that using different taxonomic levels as input would be the main factor accounting for the different outcomes because the effect of plastic types on bacterial community was also seen to be generally low in another study when looking at the genus level. However, there were still some significant differences between biofilms on diverse polymer types [48]. In our study, species was used as an input, while genus was used as such in the other study. The genus might not provide a sufficient in-depth taxonomic resolution for determining the impact of a plastic polymer type on the bacterial community. In another study, it was shown that the relative abundance in the bacterial community at the genus level was significantly different between microplastics, surrounding water, and rick microbial mats; however, it is unclear if this is also the case for different types of plastics [45]. The common observation that was seen across these studies was an increased richness in the bacterial community in microplastics compared to those in water. In South African samples, bacteria from wastewater were clustered well and separate from the rest of bacteria present on the material.

Our previous work showed that the artificial aging process changed the surface morphology of microplastics from a smooth, uniform surface to a rougher surface with many cracks and grooves (Figure S1). In this study, ultra-violet light (UV) was used to induce the photooxidative degradation process which may result in fragmentation and/or surface ablation [30], while hydrogen peroxide was used as a strong oxidizing agent to make structural changes [32]. Recent studies mainly investigated the effect of aged microplastics on bacterial community from soil, river, and coastal area. They showed that aged microplastics seemed to pose some effects on total biomass, metabolic pathway, enzyme activity, and microbial community structure [33,34,35]. Notably, it was shown that aged microplastics significantly enriched potentially human pathogenic genes [49]. Very few studies investigated the effect of aged microplastics on the bacterial community in wastewater, although it showed that wastewater treatment plants changed the morphology, sizes, and chemical properties of microplastics [50]. In our study, there was no significant effect of all aging treatments (UV and hydrogen peroxide with duration of 1 h or 24 h) on the bacterial community from inlet wastewater. However, a significant effect on bacterial community from outlet wastewater was seen when materials were treated with hydrogen peroxide for 24 h. There are a few explanations to be accounted for this difference: (1) these aging treatments were unlikely to degrade materials enough to create a significant shift in microbial community; (2) the samples were less diverse in the outlet wastewater set-up; (3) the diversity of bacterial community was also likely reduced in outlet wastewater. According to a previous study, degradation on a molecular level was only observed for polyamide based on gel permeation chromatography analysis when hydrogen peroxide was used to treat different types of microplastics [32]. A study that showed the significant aging effect on microbial community had materials exposed to UV for an extended duration of at least 15 days [49]. Also, it is important to point out that UV-A (315–400 nm) was used to age materials instead of UV-C (100–280 nm), and the aging treatment occurred at the same time as the incubation in that study.

Since the COVID-19 pandemic, wastewater-based surveillance has been emerging as an alternative way to obtain data on population-wise infectious disease, including AMR [9,51,52]. Both the *bla*_FOX_ and *bla*_MOX_ genes belong to AmpC-type β-lactamases, hydrolyse extended-spectrum cephalosporins, and cephamycins [53]. The qPCR results showed that both the *bla*_FOX_ and *bla*_MOX_ genes in the Norwegian wastewater outlet were generally lower compared to those in the inlet, while this seemed to be the opposite in South African wastewater samples. This could be due to the difference in wastewater treatment methods between these sites. In the Norwegian facility, a 300 µM mesh filter was used to remove particles larger than 300 µM from the inlet. On the other hand, in the South African treatment facility, conventional wastewater treatment is employed using mesh screens (3–12.5 mm). Although these genes were reduced in the Norwegian wastewater outlet, the abundance of these genes were much higher in the Norwegian site in comparison with the South African site. This was because the Norwegian site was receiving wastewater from the industrial area and a hospital nearby.

In recent years, metagenomic sequencing has been widely used to investigate the presence and abundance of AMR genes as well as mobile genetic elements in WWTPs [52,54,55,56]. Microorganisms were shown to form biofilm and colonize microplastic particles. Therefore, microplastics can play the role of a carrier to facilitate the horizontal transfer of AMR genes [14,16]. To date, very few studies investigated the effect of microplastics on AMR gene abundance in WWTPs, even less so for specific plastic types. In our study, the preliminary results showed that the proliferation of AMR genes were observed on most materials in both the inlet and outlet set-ups, especially more on certain microplastics. To our surprise, the abundance of AMR genes seems to only increase on certain plastic types (PE, PET, and PLA) and decrease in PVC.A compared to that of rocks or wastewater samples. This observation was seen to be similar in both the inlet and outlet set-ups. This might be due to the higher inherent toxicity of PVC.A compared to other plastic types [57,58]. PVC.A is commonly used in construction for doors, pipes, and fittings, while PET is normally used in food packaging. However, in another study, the abundance of AMR genes was higher on rocks and microplastics (PVC) compared to that in river water [59]. In that study, only a single type of microplastics (PVC) was used for testing, and all the materials were incubated in the same bioreactor. Contrasting results regarding the effect of microplastics on potential pathogenic bacteria were reported before in two independent studies [16,60]. In one study, microplastics supported the colonization of potentially pathogenic bacteria in comparison to the river-originated planktonic community [60]. Moreover, potentially pathogenic bacteria were found to be more abundant in the planktonic bacterial community in wastewater opposed to those on microplastics [16].

## 5. Conclusions

This study explored the role of different types of microplastics on the bacterial community and AMR genes. It was shown that plastic type played a pivotal role in shaping bacterial community. The *Acinetobacter* species showed a strong association with biofilm on plastic groups compared to that on rock groups. Aging treatment using ultra-violet light did not show any significant effects on bacterial community profiles, while hydrogen peroxide treatment did. AMR genes were abundantly present in the outlet, indicating that the WWTPs in this study did not effectively reduce the abundance of these genes.

To date, microplastics and antimicrobial resistance are two of the most important health-related anthropogenic pollution problems in the environment. Yet, very little practical data on the impact of microplastics on bacterial community and AMR genes were reported, even less so for plastic types. Locating the source of these problems and building better management strategies are essential in mitigating AMR threat to humans, animals, and the environment.

## Data Availability

All sequencing data are deposited in the National Center for Biotechnology Information (NCBI) Sequence Read Archive under BioProject ID PRJNA1061070.

## Supplementary Materials

The following supporting information can be downloaded at: https://www.mdpi.com/article/10.3390/microorganisms13020260/s1, Figure S1: High-resolution SEM images of HDPE, HDPS, PP, PVC A, and PVC B before and after exposure to UV irradiation, ethanol and chlorine. (A) Virgin HDPE, (B) aged HDPE, (C) virgin HDPS, (D) aged HDPS, (E) virgin PP, (F) aged PP, (G) virgin PVC A, (H) aged PVC A, (I) virgin PVC B, and (J) aged PVC B. All images were captured at 20.0 k × magnification. Text S1: Microbial species that showed a statistically significant association in each material (Black rock, White rock, HPDE, PE, PET, PVC.A, PLA)/wastewater, and their combined groups using a specific R package (indicspecies) to perform Indicator Species Analysis with Norwegian inlet wastewater set-up. Text S2: Microbial species that showed a statistically significant association in each material group (plastics, rocks or wastewater) or their combined groups using a specific R package (indicspecies) to perform Indicator Species Analysis with Norwegian outlet wastewater set-up. Text S3: Microbial species that showed a statistically significant association in each specific material (Black rock, White rock, HPDE, PE, PET, PVC.A, PLA)/wastewater and their combined groups using a specific R package (indicspecies) to perform Indicator Species Analysis with Norwegian outlet wastewater set-up. Text S4: Microbial species that showed a statistically significant association in each material group (plastics/rocks) or their combined groups using a specific R package (indicspecies) to perform Indicator Species Analysis with South African inlet and outlet wastewater set-up. Text S5: Microbial species that showed a statistically significant association in each material (Black rock, PET, PVC.A, PLA) or their combined groups using a specific R package (indicspecies) to perform Indicator Species Analysis with South African inlet and outlet wastewater set-up. Table S1: AMR genes detected using staramr tool. Table S2: Mobile genetic elements detected on chromosome using mobsuite tool. Table S3: Mobile genetic elements detected on plasmid using mobsuite tool.
