# Supplementary material for: Microplastic Categories Distinctively Impact Wastewater Bacterial Taxonomic Composition and Antimicrobial Resistance Genes
Source: Microorganisms. 2025 Jan 24;13(2):260. doi: 10.3390/microorganisms13020260 (PMC11857732; doi:10.3390/microorganisms13020260)
Supplement: Supplementary file 1 [file microorganisms-13-00260-s001.zip › microorganisms-3363520-supplementary.pdf]

Microplastic Categories Distinctively Impact Wastewater Bacterial Taxonomic Composition and Antimicrobial Resistance Genes

Tam Thanh Tran [1,*], Kabelo Stephans Stenger [2], Marte Strømmen [1], Cornelius Carlos Bezuidenhout [2] and Odd-Gunnar Wikmark [1,2]

[1] Norwegian Research Centre AS (NORCE), Nygårdstangen, 5838 Bergen, Norway; marte-strommen@live.no (M.S.); ogwi@norceresearch.no (O.-G.W.)

[2] Unit for Environmental Sciences and Management–Microbiology, North-West University, Potchefstroom 2520, South Africa; kabelostenger@gmail.com (K.S.S.); carlos.bezuidenhout@nwu.ac.za (C.C.B.)

* Correspondence: tran@norceresearch.no

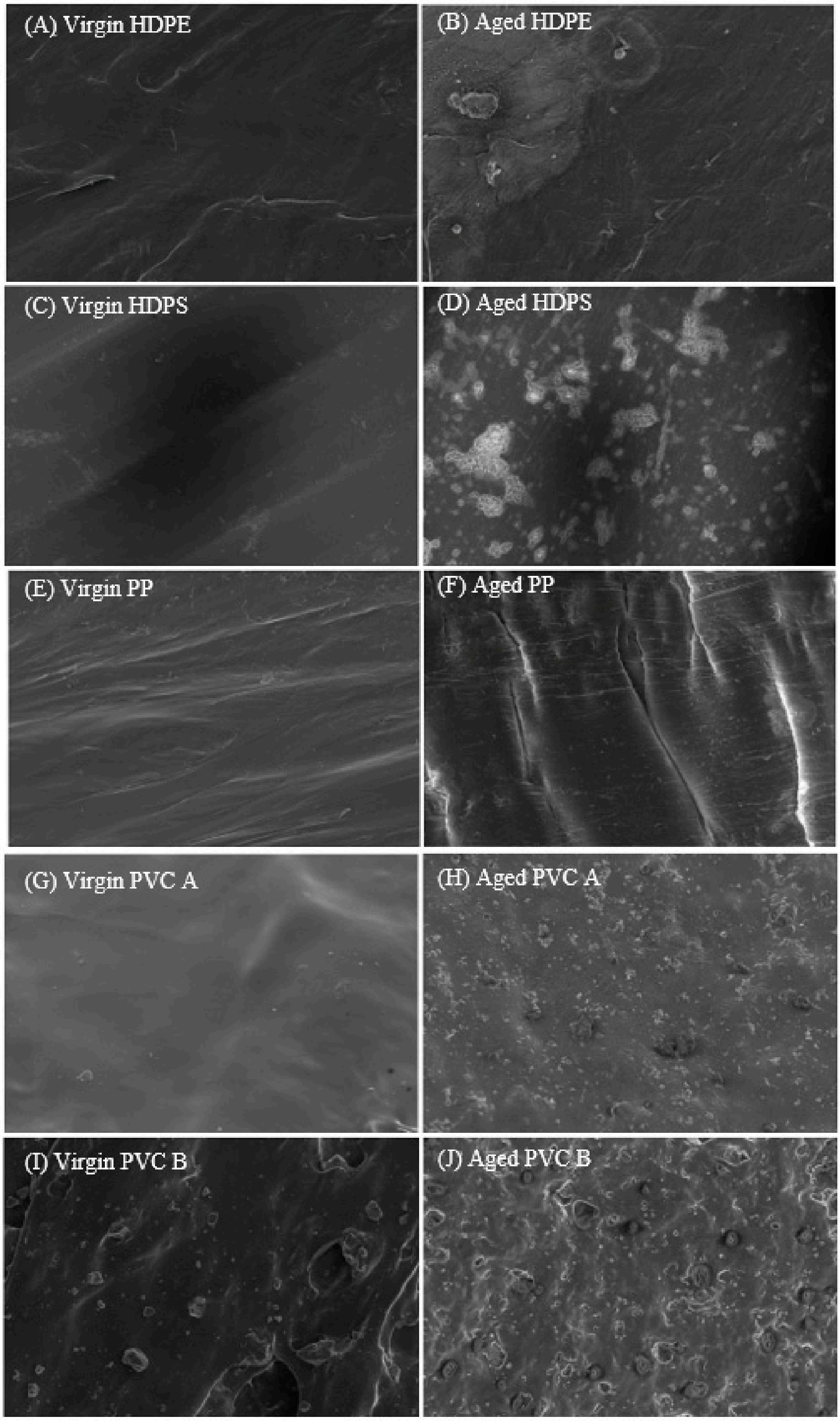

Figure S1: High-resolution SEM images of HDPE, HDPS, PP, PVC A, and PVC B before and after exposure to UV irradiation, ethanol and chlorine. (A) Virgin HDPE, (B) aged HDPE, (C) virgin HDPS, (D) aged HDPS, (E) virgin PP, (F) aged PP, (G) virgin PVC A, (H) aged PVC A, (I) virgin PVC B, and (J) aged PVC B. All images were captured at 20.0 k × magnification.

Text S1: Microbial species that showed a statistically significant association in each material (Black rock, White rock, HPDE, PE, PET, PVC.A, PLA)/wastewater, and their combined groups using a specific R package (indicspecies) to perform Indicator Species Analysis with Norwegian inlet wastewater set-up.

```
Multilevel pattern analysis
 ---------------------------

 Association function: r.g
 Significance level (alpha): 0.05

 Total number of species: 5296
 Selected number of species: 42
 Number of species associated to 1 group: 41
 Number of species associated to 2 groups: 1
 Number of species associated to 3 groups: 0
 Number of species associated to 4 groups: 0
 Number of species associated to 5 groups: 0
 Number of species associated to 6 groups: 0
 Number of species associated to 7 groups: 0

 List of species associated to each combination:

 Group PE  #sps.  10
                                     stat p.value
Pseudomonas composti                0.816  0.0102 *
Rhizobium ipomoeae                  0.798  0.0180 *
Alishewanella longhuensis           0.775  0.0114 *
Leadbetterella byssophila DSM 17132 0.736  0.0141 *
Rheinheimera tangshanensis          0.707  0.0230 *
Aliterella antarctica               0.644  0.0381 *
Alishewanella solinquinati          0.642  0.0292 *
Taishania pollutisoli               0.642  0.0299 *
Alishewanella jeotgali KCTC 22429   0.622  0.0417 *
Stagnimonas aquatica                0.615  0.0305 *

 Group PET  #sps.  2
                            stat p.value
Rhizobium arsenicireducens 0.707  0.0199 *
Flavobacterium hercynium   0.682  0.0190 *

 Group PLA  #sps.  1
                         stat p.value
Exiguobacterium marinum 0.668  0.0217 *

 Group PVC.A  #sps.  28
                                      stat p.value
Marinobacter nauticus ATCC 49840     0.723  0.0208 *
Pseudomonas alkylphenolica           0.723  0.0208 *
Pseudomonas trivialis                0.708  0.0194 *
Pseudomonas rhodesiae                0.702  0.0208 *
Yersinia frederiksenii               0.699  0.0204 *
Aeromonas tecta                      0.694  0.0195 *
Janthinobacterium svalbardensis      0.693  0.0201 *
Pelosinus defluvii                   0.692  0.0223 *
Citrobacter freundii                 0.685  0.0235 *
Janthinobacterium aquaticum          0.683  0.0191 *
Pseudomonas grimontii                0.681  0.0227 *
Psychrosinus fermentans              0.681  0.0151 *
Yersinia kristensenii                0.678  0.0188 *
Pelosinus fermentans DSM 17108       0.670  0.0178 *
Aeromonas veronii                    0.669  0.0214 *
Oceanimonas doudoroffii              0.669  0.0255 *
Pseudomonas marginalis               0.667  0.0208 *
Pseudomonas asturiensis              0.648  0.0238 *
Pseudomonas meridiana                0.647  0.0386 *
```

```
Citrobacter werkmanii                   0.639  0.0486 *
Anaerosinus glycerini                   0.638  0.0176 *
Lelliottia amnigena                     0.637  0.0364 *
Pseudomonas veronii                     0.632  0.0307 *
Pseudomonas antarctica                  0.629  0.0370 *
Yersinia aldovae                        0.625  0.0310 *
Desulfovibrio aerotolerans              0.615  0.0343 *
Aeromonas salmonicida subsp. smithia 0.613  0.0490 *
Aeromonas molluscorum                   0.603  0.0496 *

 Group PE+PET  #sps.  1
                             stat p.value
Chryseobacterium treverense 0.636  0.0382 *
---
Signif. codes:  0 ‘***’ 0.001 ‘**’ 0.01 ‘*’ 0.05 ‘.’ 0.1 ‘ ’ 1
```

Text S2: Microbial species that showed a statistically significant association in each material group (plastics, rocks or wastewater) or their combined groups using a specific R package (indicspecies) to perform Indicator Species Analysis with Norwegian outlet wastewater set-up.

```
Multilevel pattern analysis
 ---------------------------

 Association function: r.g
 Significance level (alpha): 0.05

 Total number of species: 9455
 Selected number of species: 144
 Number of species associated to 1 group: 144
 Number of species associated to 2 groups: 0

 List of species associated to each combination:

 Group Plastics  #sps.  27
                                          stat p.value
Pseudomonas anguilliseptica              0.598  0.0176 *
Stenotrophomonas acidaminiphila          0.598  0.0151 *
Sphingobium naphthae                     0.567  0.0219 *
Pseudomonas borbori                      0.540  0.0277 *
Flavobacterium acidificum                0.539  0.0282 *
Pseudomonas glareae                      0.539  0.0257 *
Shewanella oneidensis                    0.536  0.0264 *
Pseudomonas peli                         0.534  0.0454 *
Shewanella abyssi                        0.532  0.0308 *
Pseudomonas marincola                    0.525  0.0385 *
Thiomicrospira cyclica ALM1              0.523  0.0364 *
Pseudomonas helleri                      0.518  0.0496 *
Sphingobium xenophagum                   0.517  0.0364 *
Pseudomonas chlororaphis subsp. aurantiaca 0.516  0.0361 *
Xanthomonas translucens                  0.515  0.0393 *
Leucothrix mucor DSM 2157                0.513  0.0330 *
Stenotrophomonas ginsengisoli            0.512  0.0428 *
Pseudomonas extremorientalis             0.511  0.0435 *
Pseudomonas composti                     0.505  0.0432 *
Flavobacterium kingsejongi               0.504  0.0473 *
Stenotrophomonas maltophilia             0.504  0.0425 *
Sphingobium lucknowense F2               0.500  0.0438 *
Sedimenticola selenatireducens           0.492  0.0476 *
Stenotrophomonas humi                    0.487  0.0362 *
Lysobacter caeni                         0.483  0.0458 *
Janthinobacterium aquaticum              0.464  0.0473 *
Flavobacterium lindanitolerans           0.446  0.0494 *

 Group Rocks  #sps.  15
                               stat p.value
Marinomonas aquiplantarum      0.548  0.0259 *
Winogradskyella thalassocola   0.516  0.0292 *
```

Marivita roseacus	0.478	0.0476	*
Psychrobacter aestuarii	0.476	0.0310	*
Halobacteriovorax litoralis	0.470	0.0455	*
Paracoccus alkenifer	0.470	0.0478	*
Rickettsia canadensis	0.463	0.0301	*
Aquimixticola soesokkakensis	0.463	0.0472	*
Steroidobacter agariperforans	0.462	0.0421	*
Cephaloticoccus primus	0.453	0.0461	*
Fulvimonas yonginensis	0.447	0.0262	*
Thalassotalea ponticola	0.447	0.0250	*
Jeotgalibacillus marinus	0.447	0.0263	*
Anoxybacillus amylolyticus	0.447	0.0272	*
Paracholeplasma brassicae 0502	0.441	0.0495	*

Group Wastewater #sps. 102

	stat	p.value	
Legionella quateirensis	0.803	0.0030	**
Pelotomaculum thermopropionicum SI	0.765	0.0019	**
Listeria innocua	0.756	0.0038	**
Rhodococcus rhodochrous	0.721	0.0075	**
Novipirellula aureliae	0.707	0.0038	**
Leptolinea tardivitalis	0.707	0.0038	**
Yersinia nurmii	0.702	0.0030	**
Friedmanniella luteola	0.688	0.0101	*
Rhodococcus gannanensis	0.688	0.0109	*
Spirosoma jeollabukense	0.688	0.0115	*
Bacillus alkalitolerans	0.685	0.0091	**
Auraticoccus cholistanensis	0.674	0.0022	**
Nocardioides lianchengensis	0.668	0.0070	**
Auraticoccus monumenti	0.657	0.0121	*
Ornithinibacillus salinisoli	0.657	0.0128	*
Microlunatus sagamiharensis	0.645	0.0003	***
Microbacterium xylanilyticum	0.644	0.0189	*
Brevibacterium sanguinis	0.644	0.0181	*
Rhodanobacter humi	0.644	0.0189	*
Rarobacter faecitabidus	0.643	0.0109	*
Microbacterium hydrothermale	0.619	0.0144	*
Peptoniphilus coxii	0.617	0.0083	**
Roseomonas aquatica	0.612	0.0074	**
Nitrosomonas europaea	0.612	0.0050	**
Ilumatobacter fluminis YM22-133	0.604	0.0139	*
Shigella dysenteriae	0.593	0.0187	*
Arthrobacter oryzae	0.588	0.0206	*
Microbacterium mangrovi	0.588	0.0339	*
Rhodopirellula heiligendammensis	0.584	0.0126	*
Pengzhenrongella sicca	0.584	0.0144	*
Anaerofilum agile	0.583	0.0046	**
Microbacterium profundi	0.573	0.0142	*
Botrimarina mediterranea	0.567	0.0272	*
Microbacterium lacus	0.566	0.0074	**
Arthrobacter methylotrophus	0.561	0.0020	**
Nocardioides alpinus	0.556	0.0161	*
Brachybacterium avium	0.556	0.0282	*
Microlunatus spumicola	0.554	0.0024	**
Aureliella helgolandensis	0.554	0.0213	*
Egibacter rhizosphaerae	0.547	0.0345	*
Leucobacter chromiireducens	0.547	0.0099	**
Agrococcus terreus	0.545	0.0191	*
Pseudarthrobacter phenanthrenivorans Sphe3	0.545	0.0140	*
Flavimarina pacifica	0.543	0.0252	*
Tetrasphaera australiensis	0.540	0.0231	*
Serratia proteamaculans	0.539	0.0192	*
Bacillus wudalianchiensis	0.532	0.0397	*
Caloramator australicus RC3	0.530	0.0170	*
Arthrobacter pascens	0.529	0.0204	*
Caloramator indicus	0.529	0.0175	*
Polaromonas glacialis	0.522	0.0390	*
Diaminobutyricimonas aerilata	0.520	0.0276	*
Patulibacter minatonensis DSM 18081	0.519	0.0370	*
Demequina lutea	0.515	0.0264	*

```
Desulfobulbus propionicus DSM 2032          0.510  0.0124 *
Serinibacter salmoneus                      0.506  0.0160 *
Gimesia maris                               0.503  0.0474 *
Desulforhopalus singaporensis               0.502  0.0168 *
Leucobacter japonicus                       0.501  0.0342 *
Clostridium estertheticum subsp. laramiense 0.500  0.0157 *
Fonticella tunisiensis                      0.499  0.0184 *
Desulfopila inferna                         0.496  0.0265 *
Arthrobacter psychrolactophilus             0.495  0.0495 *
Sedimentibacter acidaminivorans             0.494  0.0273 *
Advenella faeciporci                        0.494  0.0294 *
Litorihabitans aurantiacus                  0.491  0.0297 *
Sedimentibacter saalensis                   0.487  0.0316 *
Demequina oxidasica                         0.487  0.0234 *
Homoserinimonas aerilata                    0.479  0.0408 *
Christensenella timonensis                  0.472  0.0314 *
Microbacterium aurum                        0.471  0.0320 *
Paenibacillus medicaginis                   0.471  0.0274 *
Tessaracoccus massiliensis                  0.471  0.0308 *
Acutalibacter muris                         0.471  0.0316 *
Arachnia rubra                              0.468  0.0360 *
Rhodococcus corynebacterioides              0.468  0.0369 *
Irregularibacter muris                      0.467  0.0368 *
Sporosalibacterium tautonense               0.467  0.0306 *
Agrococcus carbonis                         0.465  0.0219 *
Microbacterium halophytorum                 0.465  0.0414 *
Microlunatus phosphovorus NM-1              0.465  0.0418 *
Aliivibrio logei                            0.465  0.0401 *
Salinibacterium hongtaonis                  0.464  0.0442 *
Arthrobacter roseus                         0.463  0.0424 *
Rubripirellula amarantea                    0.462  0.0491 *
Nocardioides houyundeii                     0.460  0.0311 *
Caldicoprobacter faecalis                   0.452  0.0370 *
Desulfobacter postgatei                     0.449  0.0473 *
Rhabdanaerobium thermarum                   0.449  0.0379 *
Naumannella cuiyingiana                     0.448  0.0443 *
Acetonema longum DSM 6540                   0.448  0.0467 *
Terracoccus luteus                          0.448  0.0462 *
Dolosicoccus paucivorans                    0.448  0.0463 *
Georgenia soli                              0.447  0.0441 *
Desulfatibacillum alkenivorans              0.446  0.0379 *
Desulfomonile tiedjei DSM 6799              0.446  0.0414 *
Christensenella hongkongensis               0.445  0.0466 *
Saccharofermentans acetigenes               0.445  0.0490 *
Syntrophomonas palmitatica                  0.443  0.0500 *
Christensenella minuta                      0.438  0.0468 *
Glaciihabitans tibetensis                   0.434  0.0450 *
Caldicoprobacter guelmensis                 0.425  0.0484 *
---
Signif. codes:  0 ‘***’ 0.001 ‘**’ 0.01 ‘*’ 0.05 ‘.’ 0.1 ‘ ’ 1
```

Text S3: Microbial species that showed a statistically significant association in each specific material (Black rock, White rock, HPDE, PE, PET, PVC.A, PLA)/wastewater and their combined groups using a specific R package (indicspecies) to perform Indicator Species Analysis with Norwegian outlet wastewater set-up.

```
Multilevel pattern analysis
 ---------------------------

 Association function: r.g
 Significance level (alpha): 0.05

 Total number of species: 9455
 Selected number of species: 410
 Number of species associated to 1 group: 240
 Number of species associated to 2 groups: 129
 Number of species associated to 3 groups: 34
 Number of species associated to 4 groups: 7
```

```
 Number of species associated to 5 groups: 0
 Number of species associated to 6 groups: 0
 Number of species associated to 7 groups: 0

 List of species associated to each combination:

 Group BR  #sps.  12
                             stat p.value
Fulvimonas yonginensis     0.683  0.0274 *
Colwellia piezophila       0.656  0.0150 *
Paracoccus litorisediminis 0.603  0.0273 *
Oceaniferula marina        0.590  0.0230 *
Roseivirga pacifica        0.590  0.0240 *
Rhodopirellula rubra       0.579  0.0483 *
Rubripirellula tenax       0.553  0.0410 *
Paracoccus aestuariivivens 0.553  0.0360 *
Paracoccus limosus         0.549  0.0414 *
Paracoccus yeei            0.546  0.0467 *
Paracoccus denitrificans   0.545  0.0477 *
Paracoccus aminovorans     0.543  0.0488 *

 Group HPDE  #sps.  55
                                             stat p.value
Chryseobacterium scophthalmum              0.874  0.0003 ***
Pseudomonas congelans                      0.847  0.0001 ***
Rhizobium borbori                          0.847  0.0002 ***
Stenotrophomonas humi                      0.840  0.0013 **
Rhizobium paknamense                       0.808  0.0001 ***
Candidimonas humi                          0.808  0.0003 ***
Herbaspirillum autotrophicum               0.800  0.0002 ***
Vibrio aerogenes                           0.798  0.0067 **
Levilactobacillus koreensis JCM 16448      0.798  0.0085 **
Castellaniella defragrans                  0.793  0.0005 ***
Janthinobacterium violaceinigrum           0.746  0.0026 **
Sphingomicrobium astaxanthinifaciens       0.737  0.0027 **
Myroides guanonis                          0.733  0.0043 **
Stenotrophomonas pavanii                   0.723  0.0025 **
Pseudomonas antarctica                     0.720  0.0005 ***
Bordetella trematum                        0.719  0.0007 ***
Stenotrophomonas acidaminiphila            0.714  0.0022 **
Stenotrophomonas nitritireducens           0.712  0.0039 **
Pseudomonas tremae                         0.711  0.0013 **
Flavobacterium gossypii                    0.708  0.0129 *
Pseudomonas ficuserectae                   0.703  0.0021 **
Fulvimarina endophytica                    0.697  0.0030 **
Oligella urethralis                        0.697  0.0027 **
Neorhizobium huautlense                    0.696  0.0032 **
Eikenella corrodens                        0.696  0.0184 *
Oligella ureolytica DSM 18253              0.696  0.0179 *
Roseomonas suffusca                        0.696  0.0173 *
Chryseobacterium arachidis                 0.696  0.0200 *
Chryseobacterium greenlandense             0.696  0.0179 *
Vibrio ezurae                              0.696  0.0195 *
Pseudomonas meridiana                      0.691  0.0021 **
Shewanella xiamenensis                     0.677  0.0045 **
Paracandidimonas soli                      0.665  0.0046 **
Pusillimonas thiosulfatoxidans             0.657  0.0030 **
Janthinobacterium svalbardensis            0.644  0.0152 *
Deefgea rivuli                             0.641  0.0084 **
Pusillimonas caeni                         0.639  0.0057 **
Kocuria uropygioeca                        0.633  0.0244 *
Pseudomonas syringae                       0.629  0.0077 **
Aeromonas salmonicida subsp. pectinolytica 0.620  0.0120 *
Lacticaseibacillus camelliae               0.619  0.0330 *
Aliivibrio sifiae                          0.619  0.0317 *
Rhizobium glycinendophyticum               0.619  0.0101 *
Pusillimonas ginsengisoli                  0.613  0.0098 **
Rhizobium populi                           0.611  0.0138 *
Pseudomonas arsenicoxydans                 0.611  0.0128 *
Tepidimonas taiwanensis                    0.608  0.0227 *
```

Methylophaga nitratireducenticrescens	0.606	0.0376	*
Stenotrophomonas ginsengisoli	0.603	0.0144	*
Paralcaligenes ginsengisoli	0.596	0.0136	*
Pseudomonas cerasi	0.584	0.0198	*
Shewanella algae	0.584	0.0194	*
Xanthomonas axonopodis	0.571	0.0391	*
Pseudomonas grimontii	0.552	0.0406	*
Pseudomonas asplenii	0.547	0.0345	*

Group PE #sps. 13

	stat	p.value	
Alkanindiges illinoisensis	0.875	0.0003	***
Acinetobacter halotolerans	0.836	0.0003	***
Rhizobium sullae	0.798	0.0013	**
Shewanella aquimarina	0.689	0.0022	**
Mesorhizobium robiniae	0.683	0.0250	*
Microvirgula curvata	0.683	0.0250	*
Pseudomonas borbori	0.629	0.0090	**
Shewanella abyssi	0.603	0.0130	*
Herbaspirillum frisingense	0.601	0.0208	*
Pseudomonas caricapapayae	0.596	0.0180	*
Pseudomonas tolaasii NCPPB 2192	0.581	0.0233	*
Pseudomonas marincola	0.561	0.0301	*
Aeromonas allosaccharophila	0.544	0.0403	*

Group PET #sps. 32

	stat	p.value	
Cupriavidus lacunae	0.683	0.0254	*
Roseomonas aerofrigidensis	0.656	0.0182	*
Constrictibacter antarcticus	0.633	0.0138	*
Novosphingobium colocasiae	0.607	0.0147	*
Methylomonas lenta	0.601	0.0228	*
Moraxella boevrei	0.601	0.0217	*
Mesorhizobium mediterraneum	0.598	0.0347	*
Mitsuaria noduli	0.584	0.0295	*
Psychrobacillus lasiicapitis	0.578	0.0265	*
Flavitalea flava	0.571	0.0281	*
Sphingomonas koreensis	0.569	0.0322	*
Devosia chinhatensis	0.564	0.0300	*
Comamonas aquatilis	0.563	0.0290	*
Undibacterium danionis	0.562	0.0428	*
Singulisphaera acidiphila DSM 18658	0.560	0.0339	*
Inhella inkyongensis	0.560	0.0304	*
Legionella spiritensis	0.557	0.0305	*
Pseudoxanthomonas mexicana	0.555	0.0342	*
Rhodocaloribacter litoris	0.554	0.0361	*
Rugosibacter aromaticivorans	0.554	0.0300	*
Anabaena cylindrica PCC 7122	0.553	0.0455	*
Pandoraea terrae	0.553	0.0445	*
Thiomonas islandica	0.548	0.0396	*
Pseudoxanthomonas japonensis	0.546	0.0371	*
Nitrosomonas ureae	0.544	0.0260	*
Caballeronia grimmiae	0.542	0.0455	*
Sphingobium limneticum	0.541	0.0433	*
Herbaspirillum lusitanum	0.538	0.0483	*
Singulisphaera rosea	0.536	0.0365	*
Methylogaea oryzae	0.535	0.0458	*
Flavobacterium gilvum	0.534	0.0481	*
Ectothiorhodospira magna	0.523	0.0449	*

Group PLA #sps. 19

	stat	p.value	
Flavobacterium lindanitolerans	0.798	0.0006	***
Sphingomonas gotjawalisoli	0.726	0.0016	**
Alishewanella longhuensis	0.706	0.0018	**
Fangia hongkongensis	0.683	0.0267	*
Rheinheimera japonica	0.665	0.0030	**
Rheinheimera baltica	0.658	0.0121	*
Rheinheimera aquimaris	0.630	0.0137	*
Pantoea ananatis	0.605	0.0147	*

Phaseolibacter flectens ATCC 12775	0.603	0.0209	*
Vibrio halioticoli	0.601	0.0227	*
Vibrio tapetis subsp. britannicus	0.589	0.0284	*
Sphingobium naphthae	0.585	0.0215	*
Vibrio hispanicus	0.564	0.0333	*
Rheinheimera marina	0.560	0.0320	*
Pantoea cypripedii	0.557	0.0304	*
Brenneria alni	0.554	0.0241	*
Agarivorans litoreus	0.547	0.0489	*
Rheinheimera aestuarii	0.546	0.0455	*
Vibrio penaeicida	0.546	0.0416	*

Group PVC.A #sps. 75

	stat	p.value	
Brevibacterium ravenspurgense 5401308 = CCUG 53855	0.683	0.0282	*
Microlunatus endophyticus	0.683	0.0282	*
Schleiferilactobacillus similis DSM 23365 = JCM 2765	0.683	0.0282	*
Campylobacter lanienae NCTC 13004	0.669	0.0246	*
Pedobacter westerhofensis	0.660	0.0120	*
Balneicella halophila	0.660	0.0215	*
Dysgonomonas capnocytophagoides	0.642	0.0244	*
Parabacteroides goldsteinii DSM 19448 = WAL 12034	0.642	0.0254	*
Pararhodospirillum photometricum DSM 122	0.640	0.0225	*
Polaribacter marinivivus	0.630	0.0180	*
Persicobacter diffluens	0.629	0.0180	*
Hydrogenophaga caeni	0.625	0.0180	*
Acetobacterium bakii	0.624	0.0202	*
Cellulophaga lytica	0.622	0.0222	*
Anaerocella delicata	0.619	0.0178	*
Alistipes senegalensis JC50	0.615	0.0180	*
Epilithonimonas bovis DSM 19482	0.613	0.0303	*
Ancylomarina psychrotolerans	0.606	0.0187	*
Riemerella anatipestifer	0.606	0.0229	*
Xenophilus arseniciresistens	0.604	0.0123	*
Rhodospirillum rubrum ATCC 11170	0.603	0.0213	*
Abyssisolibacter fermentans	0.601	0.0239	*
Mediterranea massiliensis	0.598	0.0333	*
Marininema mesophilum	0.598	0.0242	*
Gabonia massiliensis	0.597	0.0263	*
Sphingobacterium yamdrokense	0.597	0.0139	*
Cellulomonas carbonis T26	0.592	0.0357	*
Alistipes finegoldii	0.592	0.0263	*
Alistipes shahii	0.592	0.0248	*
Alistipes putredinis	0.591	0.0256	*
Dysgonomonas gadei ATCC BAA-286	0.587	0.0212	*
Flavobacterium tegetincola	0.586	0.0235	*
Acetobacterium psammolithicum	0.584	0.0173	*
Polaribacter marinaquae	0.584	0.0259	*
Vallitalea pronyensis	0.583	0.0305	*
Draconibacterium sediminis	0.581	0.0215	*
Thiofractor thiocaminus	0.579	0.0497	*
Alistipes ihumii AP11	0.579	0.0454	*
Sanguibacter suarezii	0.579	0.0466	*
Veillonella parvula	0.579	0.0466	*
Anaeromicrobium sediminis	0.577	0.0311	*
Marinifilum albidiflavum	0.577	0.0339	*
Flavobacterium olei	0.576	0.0345	*
Alistipes timonensis JC136	0.576	0.0271	*
Draconibacterium orientale	0.575	0.0243	*
Mucilaginibacter daejeonensis	0.573	0.0305	*
[Clostridium] populeti	0.572	0.0374	*
Marinifilum fragile CECT 7942	0.570	0.0497	*
Lutibacter litoralis	0.570	0.0326	*
Sphaerochaeta pleomorpha	0.568	0.0429	*
Acidaminococcus fermentans DSM 20731	0.568	0.0489	*
Desulfitobacterium chlororespirans	0.565	0.0401	*
Flavobacterium antarcticum	0.565	0.0309	*
Phaeocystidibacter marisrubri	0.565	0.0235	*
Flavobacterium brevivitae	0.564	0.0490	*
Janibacter hoylei PVAS-1	0.562	0.0493	*

Saccharicrinis aurantiacus	0.562	0.0468	*
Williamwhitmania taraxaci	0.559	0.0368	*
Salibacter halophilus	0.558	0.0326	*
Azonexus fungiphilus	0.555	0.0325	*
Microbacter margulisiae	0.554	0.0422	*
Vulgatibacter incomptus	0.553	0.0468	*
Insolitispirillum peregrinum subsp. integrum	0.552	0.0362	*
Desulfofarcimen acetoxidans DSM 771	0.552	0.0244	*
Mariniphaga sediminis	0.551	0.0441	*
Desulfitispora elongata	0.551	0.0472	*
Owenweeksia hongkongensis	0.550	0.0492	*
[Eubacterium] siraeum	0.546	0.0388	*
Wandonia haliotis NBRC 105642	0.546	0.0468	*
Pedobacter mongoliensis	0.545	0.0209	*
Paenibacillus terreus	0.543	0.0469	*
Hydromonas duriensis	0.541	0.0447	*
Hydrogenophaga soli	0.540	0.0485	*
Sulfurospirillum multivorans DSM 12446	0.535	0.0313	*
Dokdonia lutea	0.528	0.0430	*

Group WR #sps. 2

	stat	p.value	
Rickettsia massiliae	0.574	0.0191	*
Aequoribacter fuscus	0.538	0.0412	*

Group WW #sps. 32

	stat	p.value	
Listeria innocua	0.798	0.0088	**
Novipirellula aureliae	0.753	0.0071	**
Leptolinea tardivitalis	0.753	0.0088	**
Legionella quateirensis	0.728	0.0055	**
Nocardioides lianchengensis	0.708	0.0114	*
Rhodococcus rhodochrous	0.696	0.0185	*
Pelotomaculum thermopropionicum SI	0.677	0.0042	**
Microlunatus sagamiharensis	0.674	0.0016	**
Auraticoccus cholistanensis	0.669	0.0097	**
Nitrosomonas europaea	0.646	0.0141	*
Peptoniphilus coxii	0.642	0.0128	*
Microbacterium xylanilyticum	0.619	0.0312	*
Brevibacterium sanguinis	0.619	0.0305	*
Rhodanobacter humi	0.619	0.0298	*
Spirosoma jeollabukense	0.619	0.0305	*
Yersinia nurmii	0.617	0.0088	**
Anaerofilum agile	0.613	0.0138	*
Arthrobacter methylotrophus	0.609	0.0030	**
Bacillus alkalitolerans	0.604	0.0117	*
Microlunatus spumicola	0.601	0.0046	**
Pengzhenrongella sicca	0.601	0.0228	*
Microbacterium lacus	0.599	0.0120	*
Roseomonas aquatica	0.592	0.0230	*
Serratia proteamaculans	0.571	0.0208	*
Pseudarthrobacter phenanthrenivorans Sphe3	0.563	0.0300	*
Caloramator australicus RC3	0.562	0.0365	*
Diaminobutyricimonas aerilata	0.557	0.0418	*
Agrococcus terreus	0.554	0.0444	*
Desulforhopalus singaporensis	0.553	0.0317	*
Serinibacter salmoneus	0.545	0.0397	*
Desulfobulbus propionicus DSM 2032	0.536	0.0380	*
Fonticella tunisiensis	0.530	0.0495	*

Group BR+HPDE #sps. 2

	stat	p.value	
Metabacillus fastidiosus	0.620	0.0278	*
Roseovarius aestuarii	0.541	0.0382	*

Group BR+PET #sps. 5

	stat	p.value	
Ruegeria meonggei	0.620	0.0155	*
Acinetobacter baylyi	0.579	0.0271	*
Acinetobacter kookii	0.555	0.0496	*

```
Flavihumibacter cheonanensis 0.544  0.0499 *
Cupriavidus malaysiensis     0.540  0.0461 *

 Group BR+PVC.A  #sps.  1
                          stat p.value
Microbacterium testaceum 0.591  0.0238 *

 Group BR+WW  #sps.  3
                                  stat p.value
Rhodopirellula heiligendammensis 0.687  0.0039 **
Rubripirellula amarantea         0.647  0.0084 **
Nocardioides alpinus             0.601  0.0163 *

 Group HPDE+PE  #sps.  68
                                                 stat p.value
Pseudomonas fildesensis                         0.885  0.0001 ***
Pseudomonas poae                                0.852  0.0001 ***
Pseudomonas qingdaonensis                       0.848  0.0001 ***
Pseudomonas extremaustralis 14-3                0.846  0.0003 ***
Pseudomonas veronii                             0.840  0.0002 ***
Pseudomonas cichorii                            0.832  0.0001 ***
Pseudomonas lurida                              0.829  0.0005 ***
Pseudomonas composti                            0.817  0.0003 ***
Pseudomonas extremorientalis                    0.806  0.0002 ***
Pseudomonas brenneri                            0.800  0.0003 ***
Pseudomonas chlororaphis subsp. aurantiaca      0.799  0.0002 ***
Pseudomonas rhodesiae                           0.791  0.0005 ***
Pseudomonas psychrophila                        0.790  0.0006 ***
Pseudomonas proteolytica                        0.787  0.0011 **
Pseudomonas protegens                           0.777  0.0005 ***
Pigmentiphaga aceris                            0.763  0.0015 **
Pseudomonas trivialis                           0.763  0.0012 **
Pseudomonas fluorescens                         0.752  0.0007 ***
Stenotrophomonas koreensis                      0.734  0.0004 ***
Pseudomonas viridiflava                         0.733  0.0021 **
Pseudomonas marginalis                          0.733  0.0017 **
Pseudomonas chlororaphis                        0.724  0.0011 **
Pseudomonas asturiensis                         0.721  0.0004 ***
Pseudomonas orientalis                          0.710  0.0015 **
Thiomicrospira cyclica ALM1                     0.708  0.0023 **
Pseudomonas tolaasii                            0.706  0.0016 **
Pseudomonas lactis                              0.696  0.0021 **
Pseudomonas costantinii                         0.692  0.0013 **
Pseudomonas plecoglossicida                     0.692  0.0012 **
Pigmentiphaga daeguensis                        0.686  0.0031 **
Pseudomonas weihenstephanensis                  0.684  0.0030 **
Pseudomonas anguilliseptica                     0.682  0.0033 **
Pseudomonas putida                              0.679  0.0013 **
Shewanella pealeana                             0.675  0.0034 **
Pseudomonas panacis                             0.673  0.0025 **
Pseudomonas libanensis                          0.666  0.0047 **
Pseudomonas azotoformans                        0.663  0.0028 **
Pusillimonas noertemannii                       0.663  0.0025 **
Shewanella oneidensis                           0.658  0.0040 **
Shewanella profunda                             0.644  0.0061 **
Pseudomonas agarici                             0.642  0.0035 **
Pseudomonas synxantha                           0.642  0.0073 **
Shewanella japonica                             0.633  0.0074 **
Pseudomonas abietaniphila                       0.623  0.0150 *
Pseudomonas cannabina                           0.619  0.0138 *
Paralcaligenes ureilyticus                      0.617  0.0109 *
[Haemophilus] piscium                           0.613  0.0124 *
Advenella mimigardefordensis DPN7               0.613  0.0112 *
Pseudomonas corrugata                           0.608  0.0128 *
Paenalcaligenes suwonensis                      0.607  0.0109 *
Pseudomonas fragi                               0.602  0.0110 *
Pseudomonas helleri                             0.601  0.0119 *
Pseudomonas gessardii                           0.598  0.0192 *
Shewanella gelidii                              0.594  0.0187 *
Pseudorhizobium marinum                         0.588  0.0185 *
```

```
Pseudomonas savastanoi                             0.586  0.0228 *
Pseudomonas aylmerensis                            0.584  0.0257 *
Angulomicrobium tetraedrale                        0.579  0.0354 *
Shewanella amazonensis SB2B                        0.577  0.0231 *
Pseudomonas bohemica                               0.572  0.0244 *
Neisseria chenwenguii                              0.566  0.0307 *
Shewanella seohaensis                              0.566  0.0296 *
Shewanella halifaxensis HAW-EB4                    0.562  0.0278 *
Pseudomonas chlororaphis subsp. aureofaciens       0.561  0.0243 *
Pseudomonas hutmensis                              0.555  0.0376 *
Pseudomonas brassicacearum subsp. neoaurantiaca 0.546  0.0432 *
Pectobacterium betavasculorum                      0.538  0.0490 *
Pseudomonas thivervalensis                         0.537  0.0498 *

 Group HPDE+PET  #sps.  16 
                                 stat p.value    
Stenotrophomonas tumulicola        0.723  0.0029 **
Noviherbaspirillum aurantiacum     0.681  0.0049 **
Stenotrophomonas pictorum JCM 9942 0.681  0.0045 **
Stenotrophomonas terrae            0.655  0.0077 **
Kushneria indalinina               0.620  0.0246 * 
Brevundimonas halotolerans         0.604  0.0126 * 
Herminiimonas fonticola            0.595  0.0197 * 
Janthinobacterium lividum          0.592  0.0229 * 
Herminiimonas saxobsidens          0.591  0.0220 * 
Robbsia andropogonis               0.584  0.0239 * 
Stenotrophomonas daejeonensis      0.581  0.0283 * 
Aquaspirillum arcticum             0.578  0.0298 * 
Comamonas guangdongensis           0.576  0.0246 * 
Herbaspirillum huttiense           0.566  0.0485 * 
Rhizobium azooxidifex              0.557  0.0385 * 
Comamonas testosteroni             0.533  0.0446 * 

 Group HPDE+PLA  #sps.  2 
                           stat p.value   
Flavobacterium qiangtangense 0.75  0.0012 **
Flavobacterium kingsejongi   0.64  0.0072 **

 Group HPDE+PVC.A  #sps.  3 
                          stat p.value  
Alkalicella caledoniensis 0.597  0.0209 *
Paenibacillus contaminans 0.593  0.0190 *
Heliophilum fasciatum     0.541  0.0452 *

 Group HPDE+WW  #sps.  1 
                      stat p.value  
Advenella faeciporci 0.603  0.0139 *

 Group PE+PLA  #sps.  2 
                         stat p.value  
Enterovibrio norvegicus 0.570  0.0253 *
Shewanella corallii     0.566  0.0289 *

 Group PE+PVC.A  #sps.  2 
                               stat p.value  
Nautilia nitratireducens MB-1 0.620  0.0162 *
Neisseria canis               0.617  0.0167 *

 Group PE+WW  #sps.  1 
                                     stat p.value   
Patulibacter minatonensis DSM 18081 0.644  0.0086 **

 Group PET+PLA  #sps.  10 
                                stat p.value   
Pararheinheimera texasensis     0.684  0.0028 **
Rheinheimera pacifica           0.654  0.0065 **
Rheinheimera riviphila          0.638  0.0052 **
Providencia stuartii            0.604  0.0132 * 
Brenneria populi Li et al. 2015 0.595  0.0189 * 
Rheinheimera nanhaiensis E407-8 0.580  0.0215 * 
```

```
Rheinheimera perlucida          0.567  0.0266 *
Rheinheimera hassiensis         0.562  0.0284 *
Erythrobacter colymbi           0.541  0.0479 *
Rheinheimera gaetbuli           0.537  0.0499 *

 Group PET+PVC.A  #sps.  1 
                      stat p.value  
Dechloromonas agitata 0.57  0.0311 *

 Group PET+WW  #sps.  2 
                           stat p.value   
Friedmanniella luteola    0.655  0.0070 **
Zhihengliuella salsuginis 0.579  0.0208 * 

 Group PLA+PVC.A  #sps.  1 
                            stat p.value  
Leptonema illini DSM 21528 0.591  0.0197 *

 Group PVC.A+WW  #sps.  8 
                                              stat p.value   
Rhodococcus gannanensis                      0.655  0.0072 **
Clostridium estertheticum subsp. laramiense 0.596  0.0364 * 
Auraticoccus monumenti                       0.579  0.0321 * 
Tetrasphaera australiensis                   0.577  0.0385 * 
Microbacterium profundi                      0.574  0.0360 * 
Anaerosinus glycerini                        0.573  0.0425 * 
Sporosalibacterium tautonense                0.565  0.0430 * 
Desulfoscipio gibsoniae DSM 7213             0.560  0.0469 * 

 Group WR+WW  #sps.  1 
                         stat p.value  
Entomoplasma melaleucae 0.55  0.0375 *

 Group BR+PET+PVC.A  #sps.  1 
                                 stat p.value  
Microbacterium keratanolyticum 0.577  0.0303 *

 Group HPDE+PE+PET  #sps.  20 
                                            stat p.value   
Janthinobacterium aquaticum                0.690  0.0044 **
Pseudomonas multiresinivorans              0.652  0.0036 **
Aeromonas molluscorum                      0.641  0.0065 **
Duganella levis                            0.624  0.0106 * 
Stenotrophomonas maltophilia               0.616  0.0128 * 
Janthinobacterium rivuli                   0.589  0.0230 * 
Aeromonas jandaei                          0.587  0.0175 * 
Pseudomonas silesiensis                    0.581  0.0246 * 
Shewanella violacea DSS12                  0.576  0.0255 * 
Aeromonas salmonicida subsp. achromogenes 0.574  0.0230 * 
Aeromonas salmonicida subsp. smithia       0.568  0.0280 * 
Pseudomonas lundensis                      0.564  0.0264 * 
Aeromonas encheleia                        0.558  0.0348 * 
Janthinobacterium agaricidamnosum          0.554  0.0374 * 
Shinella kummerowiae                       0.554  0.0329 * 
Lysobacter korlensis                       0.546  0.0441 * 
Photobacterium chitinilyticum              0.540  0.0443 * 
Pseudomonas lini                           0.539  0.0444 * 
Pseudomonas brassicacearum                 0.538  0.0411 * 
Aeromonas rivuli                           0.537  0.0474 * 

 Group HPDE+PE+PLA  #sps.  7 
                          stat p.value  
Shewanella colwelliana   0.620  0.0110 *
Shewanella pneumatophori 0.614  0.0110 *
Shewanella waksmanii     0.587  0.0202 *
Shewanella putrefaciens  0.580  0.0204 *
Shewanella aestuarii     0.564  0.0295 *
Photobacterium swingsii  0.561  0.0302 *
Pseudomonas peli         0.555  0.0325 *
```

```
 Group HPDE+PE+PVC.A  #sps.  2
                       stat p.value
Glaciimonas frigoris  0.560  0.0341 *
Aeromonas salmonicida 0.538  0.0462 *

 Group HPDE+PET+PLA  #sps.  3
                            stat p.value
Sphingobium xenophagum     0.639  0.0068 **
Sphingobium lucknowense F2 0.578  0.0194 *
Alishewanella tabrizica    0.565  0.0258 *

 Group HPDE+PET+WW  #sps.  1
                              stat p.value
Ornithinibacillus salinisoli 0.577  0.0323 *

 Group HPDE+PE+PET+PLA  #sps.  2
                     stat p.value
Moritella viscosa   0.554  0.0348 *
Pseudomonas cedrina 0.532  0.0496 *

 Group HPDE+PE+PET+PVC.A  #sps.  3
                          stat p.value
Caldimonas hydrothermale 0.561  0.0300 *
Acidovorax radicis N35   0.558  0.0344 *
Acidovorax soli          0.545  0.0454 *

 Group HPDE+PE+PET+WW  #sps.  1
                     stat p.value
Pseudomonas migulae 0.555   0.037 *

 Group HPDE+PE+PLA+WW  #sps.  1
                    stat p.value
Shewanella morhuae 0.577  0.0236 *
---
Signif. codes:  0 ‘***’ 0.001 ‘**’ 0.01 ‘*’ 0.05 ‘.’ 0.1 ‘ ’ 1
```

Text S4: Microbial species that showed a statistically significant association in each material group (plastics/rocks) or their combined groups using a specific R package (indicspecies) to perform Indicator Species Analysis with South African inlet and outlet wastewater set-up.

```
 Multilevel pattern analysis
 ---------------------------

 Association function: r.g
 Significance level (alpha): 0.05

 Total number of species: 6469
 Selected number of species: 473
 Number of species associated to 1 group: 473

 List of species associated to each combination:

 Group Plastics  #sps.  101
                                                         stat p.value
Pseudomonas lalkuanensis                                0.562  0.0068 **
Citrobacter gillenii                                    0.548  0.0043 **
Aeromonas enteropelogenes                               0.536  0.0037 **
Acinetobacter radioresistens                            0.535  0.0067 **
Citrobacter murliniae                                   0.529  0.0018 **
Giesbergeria voronezhensis                              0.512  0.0090 **
Enterobacter sichuanensis                               0.510  0.0069 **
Acinetobacter septicus                                  0.499  0.0077 **
Klebsiella pasteurii                                    0.491  0.0059 **
Tessaracoccus flavescens                                0.489  0.0084 **
Enterobacter quasiroggenkampii                          0.487  0.0094 **
Comamonas fluminis                                      0.486  0.0122 *
Acinetobacter guillouiae                                0.485  0.0058 **
Enterobacter chengduensis                               0.481  0.0223 *
Brucella oryzae                                         0.479  0.0155 *
Acinetobacter gyllenbergii                              0.477  0.0170 *
Acinetobacter wuhouensis                                0.469  0.0067 **
```

Acinetobacter baumannii	0.468	0.0061	**
Delftia deserti	0.468	0.0136	*
Shinella pollutisoli	0.460	0.0148	*
Enterobacter wuhouensis	0.458	0.0070	**
Acinetobacter equi	0.456	0.0129	*
Rhodoferax ferrireducens T118	0.456	0.0196	*
Acinetobacter refrigeratoris	0.454	0.0121	*
Acinetobacter gandensis	0.453	0.0027	**
Roseomonas cervicalis	0.452	0.0128	*
Acinetobacter tianfuensis	0.450	0.0173	*
Rhizobium pseudoryzae	0.450	0.0133	*
Giesbergeria sinuosa	0.449	0.0181	*
Brevundimonas balnearis	0.447	0.0197	*
Phreatobacter cathodiphilus	0.447	0.0317	*
Brevundimonas mongoliensis	0.446	0.0235	*
Acinetobacter tandoii	0.446	0.0151	*
Acinetobacter baylyi	0.444	0.0217	*
Nitratireductor arenosus	0.442	0.0160	*
Ramlibacter algicola	0.442	0.0260	*
Comamonas suwonensis	0.441	0.0196	*
Caulobacter endophyticus	0.439	0.0239	*
Comamonas aquatilis	0.437	0.0102	*
Acinetobacter junii	0.437	0.0169	*
Luteimonas salinisoli	0.436	0.0309	*
Paracoccus siganidrum	0.436	0.0473	*
Acinetobacter kanungonis	0.435	0.0159	*
Acinetobacter shaoyimingii	0.435	0.0147	*
Neorhizobium petrolearium	0.430	0.0197	*
Acinetobacter variabilis	0.430	0.0144	*
Acinetobacter modestus	0.429	0.0242	*
Acinetobacter rongchengensis	0.427	0.0176	*
Pseudomonas flavescens	0.425	0.0468	*
Acinetobacter calcoaceticus	0.424	0.0244	*
Citrobacter braakii	0.420	0.0125	*
Brevundimonas canariensis	0.418	0.0179	*
Aeromonas veronii	0.417	0.0294	*
Acinetobacter indicus	0.417	0.0233	*
Rhizobium paknamense	0.416	0.0368	*
Acinetobacter bouvetii	0.416	0.0192	*
Citrobacter europaeus	0.413	0.0044	**
Brevundimonas diminuta ATCC 11568	0.413	0.0114	*
Acinetobacter lanii	0.412	0.0295	*
Vagococcus fluvialis	0.409	0.0202	*
Comamonas phosphati	0.408	0.0367	*
Brevundimonas terrae	0.406	0.0060	**
Acinetobacter johnsonii	0.403	0.0191	*
Acinetobacter indicus CIP 110367	0.401	0.0437	*
Acinetobacter vivianii	0.401	0.0405	*
Lampropedia hyalina	0.401	0.0462	*
Brevundimonas viscosa	0.400	0.0324	*
Agrobacterium leguminum	0.399	0.0483	*
Rhizobium borbori	0.399	0.0436	*
Acinetobacter albensis	0.397	0.0336	*
Pseudocitrobacter vendiensis	0.397	0.0428	*
Citrobacter freundii ATCC 8090 = MTCC 1658 = NBRC 12681	0.395	0.0083	**
Comamonas koreensis	0.395	0.0387	*
Acinetobacter chengduensis	0.394	0.0267	*
Acinetobacter oryzae	0.393	0.0225	*
Acinetobacter pittii DSM 21653	0.393	0.0392	*
Acinetobacter haemolyticus	0.392	0.0388	*
Acinetobacter nectaris CIP 110549	0.391	0.0495	*
Acinetobacter seohaensis	0.389	0.0352	*
Acinetobacter bereziniae	0.388	0.0348	*
Mycoplana ramosa	0.388	0.0351	*
Brevundimonas subvibrioides ATCC 15264	0.387	0.0222	*
Vagococcus carniphilus	0.383	0.0174	*
Comamonas aquatica subsp. rana	0.383	0.0486	*
Acinetobacter courvalinii	0.381	0.0447	*
Acinetobacter schindleri	0.379	0.0326	*
Brevundimonas diminuta	0.376	0.0303	*
Brevundimonas bullata	0.374	0.0175	*
Acinetobacter defluvii	0.374	0.0144	*
Pseudenterobacter timonensis	0.372	0.0314	*
Enterococcus hirae ATCC 9790	0.371	0.0338	*
Acinetobacter oleivorans	0.368	0.0495	*
Citrobacter pasteurii	0.366	0.0379	*
Clostridium sulfidigenes	0.363	0.0369	*

Timonella senegalensis JC301	0.362	0.0378	*
Chelatococcus daeguensis	0.361	0.0397	*
Clostridium subterminale	0.348	0.0192	*
Brevundimonas lutea	0.336	0.0392	*
Camelimonas fluminis	0.326	0.0417	*
Acinetobacter gerneri DSM 14967 = CIP 107464 = MTCC 9824	0.299	0.0443	*
Cloacibacterium haliotis	0.299	0.0433	*

Group Rocks #sps. 372

	stat	p.value	
Perlabentimonas gracilis	0.753	0.0002	***
Thermosinus carboxydivorans	0.744	0.0002	***
Kiritimatiella glycovorans	0.738	0.0001	***
Cloacibacillus porcorum	0.723	0.0001	***
Maribellus luteus	0.722	0.0001	***
Aminivibrio pyruvatiphilus	0.714	0.0001	***
Maribellus maritimus	0.687	0.0001	***
Acidaminococcus timonensis	0.678	0.0001	***
Solidesulfovibrio magneticus RS-1	0.676	0.0001	***
Cloacibacillus evryensis	0.660	0.0001	***
Geothrix fermentans	0.653	0.0002	***
Desulfomicrobium baculatum DSM 4028	0.648	0.0001	***
Propionispora vibrioides	0.645	0.0005	***
Robertkochia solimangrovi	0.631	0.0005	***
Desulfomicrobium norvegicum	0.629	0.0001	***
Alistipes finegoldii	0.627	0.0001	***
Paludibacterium purpuratum	0.626	0.0001	***
Desulfobulbus elongatus	0.619	0.0002	***
Zavarzinia compransoris	0.617	0.0001	***
Desulfuromonas michiganensis	0.612	0.0006	***
Sporomusa malonica	0.611	0.0004	***
Desulfobulbus propionicus DSM 2032	0.611	0.0003	***
Thermanaerovibrio velox	0.603	0.0001	***
Solobacterium moorei	0.581	0.0004	***
Bacteroides fluxus YIT 12057	0.579	0.0002	***
Geobacter pickeringii	0.577	0.0006	***
Tardibacter chloracetimidivorans	0.577	0.0006	***
Thermanaerovibrio acidaminovorans DSM 6589	0.570	0.0003	***
Acetonema longum DSM 6540	0.566	0.0004	***
Phocaeicola sartorii JCM 16497	0.561	0.0003	***
Anaeroarcus burkinensis	0.561	0.0014	**
Desulfomicrobium hypogeium	0.559	0.0004	***
Lentimicrobium saccharophilum	0.556	0.0002	***
Alistipes timonensis JC136	0.555	0.0010	***
Dongia mobilis	0.555	0.0019	**
Meniscus glaucopis	0.554	0.0011	**
Aminomonas paucivorans DSM 12260	0.553	0.0010	***
Veillonella infantium	0.550	0.0012	**
Megasphaera indica	0.550	0.0018	**
Anoxybacter fermentans	0.549	0.0003	***
Acidaminococcus provencensis	0.548	0.0030	**
Pusillimonas caeni	0.547	0.0002	***
Empedobacter falsenii genomovar 1	0.545	0.0024	**
Desulfovibrio porci	0.543	0.0009	***
Reyranella massiliensis 521	0.543	0.0005	***
Sphingomonas prati	0.542	0.0008	***
Alistipes shahii	0.539	0.0002	***
Kordiimonas marina	0.539	0.0009	***
Lacunisphaera limnophila	0.539	0.0010	***
Paracoccus sediminilitoris	0.539	0.0006	***
Williamwhitmania taraxaci	0.536	0.0010	***
Fusobacterium equinum	0.534	0.0018	**
Anaerospora hongkongensis	0.530	0.0015	**
Mucinivorans hirudinis	0.529	0.0012	**
Eubacterium sulci ATCC 35585	0.529	0.0030	**
Alistipes shahii WAL 8301	0.527	0.0010	***
Maribellus comscasis	0.526	0.0013	**
Solidesulfovibrio carbinolicus	0.522	0.0017	**
Verticiella sediminum	0.518	0.0004	***
Ancylobacter rudongensis	0.510	0.0039	**
Sphingobacterium faecale	0.510	0.0005	***
Fretibacterium fastidiosum	0.510	0.0013	**
Desulfonatronum cooperativum	0.509	0.0011	**
Peptoclostridium acidaminophilum	0.507	0.0013	**
Horticoccus luteus	0.504	0.0027	**
Advenella mandrilli	0.503	0.0030	**
Psychrosinus fermentans	0.499	0.0015	**

Prolixibacter denitrificans	0.498	0.0011	**
Lysobacter concretionis	0.496	0.0171	*
Roseimarinus sediminis	0.496	0.0006	***
Paludibacter propionicigenes WB4	0.494	0.0019	**
Thalassospira australica	0.491	0.0036	**
Paracandidimonas soli	0.490	0.0034	**
Desulfobulbus alkaliphilus	0.488	0.0036	**
Alistipes montrealensis	0.487	0.0046	**
Anaerosporomusa subterranea	0.485	0.0074	**
Methylobacillus glycogenes	0.483	0.0049	**
Desulfoprunum benzoelyticum	0.480	0.0059	**
Lachnoclostridium pacaense	0.480	0.0048	**
Streptococcus varani	0.480	0.0067	**
Desulfovibrio intestinalis	0.480	0.0017	**
Veillonella magna	0.480	0.0014	**
Alkaliflexus imshenetskii	0.477	0.0051	**
Aquabacter cavernae	0.477	0.0045	**
Elstera cyanobacteriorum	0.475	0.0013	**
Castellaniella denitrificans	0.475	0.0135	*
Pseudostreptobacillus hongkongensis	0.472	0.0070	**
Ralstonia solanacearum	0.470	0.0086	**
Castellaniella defragrans	0.470	0.0042	**
Propionivibrio limicola	0.469	0.0154	*
Cephaloticoccus capnophilus	0.469	0.0062	**
Geoalkalibacter ferrihydriticus	0.469	0.0062	**
Legionella hackeliae	0.469	0.0063	**
Achromobacter aloeverae	0.468	0.0135	*
Holophaga foetida	0.468	0.0018	**
Pusillimonas soli	0.468	0.0004	***
Kaistia hirudinis	0.467	0.0023	**
Aquimonas voraii	0.467	0.0030	**
Prolixibacter bellariivorans	0.466	0.0024	**
Propionicimonas paludicola	0.466	0.0110	*
Prosthecochloris marina	0.465	0.0010	***
Castellaniella hirudinis	0.464	0.0313	*
Kaistia geumhonensis	0.462	0.0011	**
Eoetvoesia caeni	0.460	0.0220	*
Thiovirga sulfuroxydans	0.460	0.0002	***
Azospirillum griseum	0.458	0.0069	**
Opitutus terrae PB90-1	0.458	0.0064	**
Sunxiuqinia rutila	0.458	0.0059	**
Aminobacterium colombiense	0.458	0.0067	**
Pollutimonas nitritireducens	0.458	0.0034	**
Veillonella atypica	0.458	0.0047	**
Acetobacteroides hydrogenigenes	0.456	0.0082	**
Mangrovibacterium marinum	0.455	0.0020	**
Propionivibrio dicarboxylicus	0.454	0.0104	*
Dorea phocaeensis	0.454	0.0075	**
Lactivibrio alcoholicus	0.453	0.0036	**
Tahibacter caeni	0.452	0.0043	**
Helcococcus sueciensis	0.451	0.0080	**
Legionella tunisiensis	0.450	0.0013	**
Labilibacter sediminis	0.450	0.0071	**
Caenibius tardaugens	0.450	0.0045	**
Halovulum marinum	0.447	0.0049	**
Millionella massiliensis	0.447	0.0067	**
Rhodopseudomonas palustris	0.447	0.0049	**
Caenimicrobium hargitense	0.444	0.0014	**
Fermentimonas caenicola	0.443	0.0209	*
Pusillimonas thiosulfatoxidans	0.442	0.0010	***
Legionella qingyii	0.442	0.0004	***
Carboxylicivirga mesophila	0.442	0.0300	*
Legionella pneumophila subsp. fraseri	0.440	0.0046	**
Reyranella aquatilis	0.439	0.0025	**
Limisphaera ngatamarikiensis	0.438	0.0034	**
Achromobacter animicus	0.437	0.0062	**
Parapusillimonas granuli	0.436	0.0029	**
Pusillimonas ginsengisoli	0.435	0.0010	***
Ampullimonas aquatilis	0.435	0.0044	**
Legionella drozanskii	0.435	0.0030	**
Rothia endophytica	0.435	0.0192	*
Alsobacter metallidurans	0.434	0.0120	*
Legionella taurinensis	0.434	0.0049	**
Desulfuromonas svalbardensis	0.432	0.0138	*
Mangrovibacterium lignilyticum	0.432	0.0119	*
Syntrophomonas bryantii	0.432	0.0124	*
Ottowia beijingensis	0.432	0.0249	*

Flavobacterium celericrescens	0.430	0.0024	**
Ignavibacterium album JCM 16511	0.430	0.0068	**
Bifidobacterium adolescentis ATCC 15703	0.430	0.0232	*
Mariniphaga sediminis	0.428	0.0173	*
Anaeromusa acidaminophila	0.427	0.0463	*
Pusillimonas minor	0.426	0.0009	***
Achromobacter xylosoxidans	0.426	0.0182	*
Magnetospira thiophila	0.426	0.0197	*
Pedococcus badiiscoriae	0.426	0.0210	*
Petrimonas mucosa	0.424	0.0108	*
Bacteroides ihuae	0.424	0.0088	**
Thermanaeromonas burensis	0.423	0.0061	**
Flavobacterium cucumis	0.419	0.0089	**
Chelativorans alearense	0.418	0.0165	*
Sphingosinicella xenopeptidilytica	0.417	0.0114	*
Azovibrio restrictus	0.417	0.0043	**
Xinfangfangia soli	0.416	0.0305	*
Legionella norrlandica	0.416	0.0008	***
Bordetella avium	0.414	0.0119	*
Oleisolibacter albus	0.414	0.0081	**
Solidesulfovibrio aerotolerans	0.413	0.0162	*
Pelistega ratti	0.411	0.0136	*
Basilea psittacipulmonis DSM 24701	0.409	0.0211	*
Azonexus hydrophilus DSM 23864	0.408	0.0114	*
Legionella santicrucis	0.407	0.0016	**
Bacteroides graminisolvens	0.407	0.0036	**
Gemmobacter aquaticus	0.406	0.0341	*
Bacteroides sedimenti	0.406	0.0163	*
Rossellomorea arthrocnemi	0.406	0.0152	*
Pusillimonas maritima	0.405	0.0076	**
Paracandidimonas caeni	0.405	0.0013	**
Melioribacter roseus P3M-2	0.403	0.0064	**
Thalassospira lohafexi	0.403	0.0127	*
Prosthecobacter fluviatilis	0.403	0.0361	*
Breznakibacter xylanolyticus	0.402	0.0362	*
Silanimonas mangrovi AK13	0.401	0.0410	*
Gemmobacter aquatilis	0.400	0.0163	*
Comamonas serinivorans	0.400	0.0350	*
Achromobacter dolens	0.400	0.0070	**
Faecalibacterium duncaniae	0.399	0.0428	*
Hydrogenoanaerobacterium saccharovorans	0.398	0.0108	*
Legionella massiliensis	0.398	0.0109	*
Pirellula staleyi DSM 6068	0.398	0.0130	*
Legionella saoudiensis	0.397	0.0057	**
Orrella dioscoreae	0.397	0.0084	**
Oleomonas sagaranensis	0.397	0.0142	*
Rhizobium tropici CIAT 899	0.397	0.0244	*
Pseudomonas linyingensis	0.396	0.0084	**
Alistipes putredinis	0.394	0.0070	**
Paenalcaligenes niemegkensis	0.394	0.0198	*
Capillibacterium thermochitinicola	0.393	0.0215	*
Pelistega europaea	0.393	0.0243	*
Pseudahrensia todarodis	0.393	0.0226	*
Traorella massiliensis	0.393	0.0199	*
Desulfobulbus rhabdoformis	0.393	0.0161	*
Phocaeicola coprophilus	0.391	0.0130	*
Pygmaiobacter massiliensis	0.390	0.0176	*
Chryseobacterium reticulitermitis	0.389	0.0185	*
Sporomusa aerivorans	0.388	0.0149	*
Legionella sainthelensi	0.386	0.0207	*
Solidesulfovibrio marrakechensis	0.386	0.0219	*
Bacteroides uniformis	0.385	0.0064	**
Agrobacterium rhizogenes	0.385	0.0479	*
Sporolituus thermophilus DSM 23256	0.385	0.0143	*
Bordetella petrii	0.385	0.0180	*
Leucobacter salsicius M1-8	0.384	0.0264	*
Hydromonas duriensis	0.384	0.0325	*
Sporomusa acidovorans	0.383	0.0121	*
Legionella rubrilucens	0.383	0.0118	*
Achromobacter denitrificans	0.382	0.0235	*
Advenella kashmirensis	0.381	0.0330	*
Irregularibacter muris	0.381	0.0252	*
Tuwongella immobilis	0.381	0.0129	*
Niveispirillum fermenti	0.380	0.0295	*
Pseudomonas glareae	0.379	0.0174	*
Wielerella bovis	0.378	0.0209	*
Candidimonas humi	0.378	0.0283	*

Alteriqipengyuania halimionae	0.378	0.0396	*
Amaricoccus macauensis	0.378	0.0360	*
Anaerotignum lactatifermentans	0.378	0.0373	*
Anaerovibrio lipolyticus DSM 3074	0.378	0.0386	*
Bacteroides faecichinchillae JCM 17102	0.378	0.0360	*
Bradyrhizobium canariense	0.378	0.0372	*
Caecibacteroides pullorum	0.378	0.0365	*
Duganella alba	0.378	0.0381	*
Flavobacterium gillisiae	0.378	0.0386	*
Hypericibacter terrae	0.378	0.0403	*
Hyphobacterium vulgare	0.378	0.0386	*
Kangiella profundi	0.378	0.0361	*
Legionella steelei	0.378	0.0365	*
Luteitalea pratensis	0.378	0.0386	*
Mariniflexile maritimum	0.378	0.0373	*
Marispirillum indicum	0.378	0.0392	*
Mogibacterium neglectum	0.378	0.0372	*
Natronincola histidinovorans	0.378	0.0376	*
Pukyongia salina	0.378	0.0360	*
Rhodococcus rhodnii	0.378	0.0360	*
Seonamhaeicola marinus	0.378	0.0361	*
Sphingobium subterraneum	0.378	0.0389	*
Sphingomonas edaphi	0.378	0.0389	*
Sunxiuqinia elliptica	0.378	0.0365	*
Thauera sinica	0.378	0.0360	*
[Eubacterium] infirmum	0.378	0.0410	*
Puteibacter caeruleilacunae	0.378	0.0062	**
Oceanibaculum pacificum	0.378	0.0186	*
Curvibacter fontanus	0.375	0.0464	*
Desulfovibrio simplex	0.375	0.0377	*
Staphylococcus simulans	0.374	0.0360	*
Advenella faeciporci	0.373	0.0083	**
Singulisphaera rosea	0.371	0.0195	*
Vescimonas coprocola	0.370	0.0203	*
Afipia carboxidovorans	0.370	0.0360	*
Gordonia alkaliphila	0.370	0.0373	*
Tautonia sociabilis	0.370	0.0361	*
Achromobacter insuavis	0.368	0.0092	**
Laribacter hongkongensis	0.368	0.0135	*
Rhodanobacter denitrificans	0.367	0.0265	*
Paludisphaera soli	0.366	0.0105	*
Acholeplasma vituli	0.366	0.0360	*
Desulfobulbus oligotrophicus	0.366	0.0376	*
Flavobacterium psychrotolerans	0.363	0.0213	*
Imtechella halotolerans	0.363	0.0209	*
Pseudoleptotrichia goodfellowii DSM 19756	0.363	0.0214	*
Aliarcobacter trophiarum LMG 25534	0.362	0.0287	*
Hyphomonas polymorpha	0.361	0.0172	*
Desulfocastanea catecholica	0.360	0.0386	*
Acetobacterium carbinolicum	0.356	0.0410	*
Alistipes provencensis	0.356	0.0410	*
Alterococcus agarolyticus	0.356	0.0376	*
Apibacter raozihei	0.356	0.0386	*
Arcobacter vandammei	0.356	0.0373	*
Bartonella gabonensis	0.356	0.0360	*
Clostridium thailandense	0.356	0.0380	*
Coraliomargarita akajimensis	0.356	0.0379	*
Desulfogranum mediterraneum	0.356	0.0410	*
Desulfopila inferna	0.356	0.0386	*
Desulforhopalus singaporensis	0.356	0.0386	*
Donghicola mangrovi	0.356	0.0365	*
Eisenbergiella porci	0.356	0.0373	*
Flavobacterium columnare	0.356	0.0360	*
Flavobacterium kingsejongi	0.356	0.0365	*
Hungatella hathewayi	0.356	0.0386	*
Labrys wisconsinensis	0.356	0.0386	*
Litorimonas haliclonae	0.356	0.0410	*
Mangrovitalea sediminis	0.356	0.0360	*
Methylotenera mobilis JLW8	0.356	0.0360	*
Motilibacter peucedani	0.356	0.0386	*
Paraburkholderia xenovorans LB400	0.356	0.0360	*
Phaeocystidibacter marisrubri	0.356	0.0376	*
Pseudomonas cichorii	0.356	0.0373	*
Pseudomonas orientalis	0.356	0.0373	*
Qipengyuania aerophila	0.356	0.0360	*
Reyranella graminifolii	0.356	0.0386	*
Rhodoplanes elegans	0.356	0.0392	*

```
Ruminiclostridium hungatei                            0.356  0.0389 *
Sphingomonas piscis                                   0.356  0.0378 *
Sunxiuqinia faeciviva                                 0.356  0.0386 *
Thermaurantimonas aggregans                           0.356  0.0373 *
Thermodesulfomicrobium thermophilum                   0.356  0.0376 *
Thiobacillus sajanensis                               0.356  0.0386 *
Skermanella aerolata                                  0.355  0.0481 *
Achromobacter piechaudii                              0.352  0.0154 *
Rhizobium croatiense                                  0.352  0.0355 *
Bacteroides cellulosilyticus                          0.352  0.0295 *
Aquihabitans daechungensis                            0.350  0.0373 *
Aromatoleum aromaticum EbN1                           0.350  0.0380 *
Geomobilimonas luticola                               0.350  0.0410 *
Gudongella oleilytica                                 0.350  0.0383 *
Lactobacillus amylovorus DSM 20531                    0.350  0.0365 *
Marinilabilia rubra                                   0.350  0.0364 *
Methylorubrum suomiense                               0.350  0.0380 *
Oryzihumus leptocrescens                              0.350  0.0360 *
Pseudomonas gessardii                                 0.350  0.0361 *
Dokdonia aurantiaca                                   0.349  0.0373 *
Legionella septentrionalis                            0.349  0.0361 *
Sphingomonas arantia                                  0.349  0.0360 *
Macromonas bipunctata                                 0.348  0.0171 *
Pusillimonas noertemannii                             0.348  0.0191 *
Legionella rowbothamii                                0.348  0.0361 *
Acidaminococcus intestini                             0.345  0.0277 *
Bacteroides rodentium JCM 16496                       0.345  0.0476 *
Elstera litoralis                                     0.344  0.0252 *
Sphingomonas lenta                                    0.344  0.0219 *
Thioclava nitratireducens                             0.344  0.0215 *
Fluviibacter phosphoraccumulans                       0.342  0.0152 *
Alistipes senegalensis JC50                           0.342  0.0123 *
Legionella lytica                                     0.342  0.0239 *
Succiniclasticum ruminis                              0.340  0.0402 *
Anaerovibrio slackiae                                 0.333  0.0360 *
Bacteroides ovatus                                    0.333  0.0386 *
Rhizorhabdus phycosphaerae                            0.333  0.0360 *
Ercella succinigenes                                  0.333  0.0481 *
Novosphingobium naphthalenivorans                     0.333  0.0477 *
Legionella longbeachae                                0.332  0.0170 *
Alysiella filiformis                                  0.331  0.0380 *
Isosphaera pallida                                    0.331  0.0383 *
Flavobacterium columnare NBRC 100251 = ATCC 23463 0.331  0.0302 *
Aliarcobacter cibarius                                0.330  0.0142 *
Gemmobacter caeni                                     0.330  0.0196 *
Oceanibaculum nanhaiense                              0.329  0.0409 *
Paludisphaera borealis                                0.328  0.0315 *
Phycicoccus duodecadis                                0.328  0.0352 *
Tautonia plasticadhaerens                             0.327  0.0492 *
Desulfovibrio vulgaris                                0.325  0.0352 *
Paracoccus shandongensis                              0.324  0.0493 *
Qipengyuania oceanensis                               0.324  0.0479 *
Aromatoleum bremense                                  0.323  0.0421 *
Rivicola pingtungensis                                0.323  0.0365 *
Bacteriovorax stolpii                                 0.318  0.0388 *
Brassicibacter thermophilus                           0.318  0.0425 *
Mannheimia ruminalis                                  0.318  0.0444 *
Kerstersia similis                                    0.314  0.0428 *
Sporomusa paucivorans                                 0.314  0.0327 *
Legionella waltersii                                  0.313  0.0181 *
Gemmobacter lanyuensis                                0.312  0.0456 *
Sphingosinicella soli                                 0.312  0.0495 *
Flavobacterium cheniae                                0.311  0.0375 *
Flavobacterium anseonense                             0.308  0.0360 *
Flavobacterium proteolyticum                          0.302  0.0203 *
Stella humosa                                         0.301  0.0326 *
Aquisphaera giovannonii                               0.297  0.0339 *
Collinsella bouchesdurhonensis                        0.295  0.0462 *
Rhodocaloribacter litoris                             0.295  0.0500 *
Anaerofustis stercorihominis                          0.291  0.0480 *
Flavobacterium buctense                               0.291  0.0482 *
Legionella fallonii                                   0.290  0.0497 *
Legionella shakespearei                               0.283  0.0200 *
Flavobacterium dauae                                  0.262  0.0392 *
---
Signif. codes:  0 ‘***’ 0.001 ‘**’ 0.01 ‘*’ 0.05 ‘.’ 0.1 ‘ ’ 1
```

Text S5: Microbial species that showed a statistically significant association in each material (Black rock, PET, PVC.A, PLA) or their combined groups using a specific R package (indicspecies) to perform Indicator Species Analysis with South African inlet and outlet wastewater set-up.

```
 Multilevel pattern analysis
 ---------------------------

 Association function: r.g
 Significance level (alpha): 0.05

 Total number of species: 6469
 Selected number of species: 496 
 Number of species associated to 1 group: 373 
 Number of species associated to 2 groups: 117 
 Number of species associated to 3 groups: 6 

 List of species associated to each combination: 

 Group BR  #sps.  202 
                                           stat p.value    
Perlabentimonas gracilis                  0.777  0.0001 ***
Maribellus luteus                         0.772  0.0001 ***
Cloacibacillus porcorum                   0.771  0.0001 ***
Kiritimatiella glycovorans                0.768  0.0001 ***
Aminivibrio pyruvatiphilus                0.761  0.0001 ***
Acidaminococcus timonensis                0.719  0.0001 ***
Cloacibacillus evryensis                  0.715  0.0001 ***
Geothrix fermentans                       0.710  0.0002 ***
Solidesulfovibrio magneticus RS-1         0.706  0.0002 ***
Paludibacterium purpuratum                0.701  0.0002 ***
Thermosinus carboxydivorans               0.682  0.0003 ***
Desulfomicrobium baculatum DSM 4028       0.682  0.0004 ***
Zavarzinia compransoris                   0.678  0.0002 ***
Thermanaerovibrio velox                   0.676  0.0002 ***
Desulfomicrobium norvegicum               0.674  0.0001 ***
Desulfuromonas michiganensis              0.665  0.0005 ***
Geobacter pickeringii                     0.655  0.0021 ** 
Tardibacter chloracetimidivorans          0.655  0.0018 ** 
Desulfobulbus elongatus                   0.650  0.0006 ***
Propionispora vibrioides                  0.649  0.0005 ***
Desulfobulbus propionicus DSM 2032        0.648  0.0003 ***
Thermanaerovibrio acidaminovorans DSM 6589 0.640  0.0001 ***
Maribellus maritimus                      0.638  0.0003 ***
Solobacterium moorei                      0.631  0.0002 ***
Lentimicrobium saccharophilum             0.630  0.0001 ***
Alistipes finegoldii                      0.620  0.0004 ***
Kordiimonas marina                        0.617  0.0013 ** 
Lacunisphaera limnophila                  0.617  0.0016 ** 
Paracoccus sediminilitoris                0.617  0.0019 ** 
Phocaeicola sartorii JCM 16497            0.615  0.0010 ***
Reyranella massiliensis 521               0.613  0.0005 ***
Sporomusa malonica                        0.612  0.0009 ***
Aminomonas paucivorans DSM 12260          0.611  0.0006 ***
Alistipes shahii                          0.606  0.0006 ***
Meniscus glaucopis                        0.605  0.0007 ***
Anoxybacter fermentans                    0.603  0.0007 ***
Robertkochia solimangrovi                 0.598  0.0010 ***
Acetonema longum DSM 6540                 0.595  0.0018 ** 
Alistipes timonensis JC136                0.592  0.0018 ** 
Williamwhitmania taraxaci                 0.590  0.0007 ***
Bacteroides fluxus YIT 12057              0.589  0.0018 ** 
Fusobacterium equinum                     0.589  0.0042 ** 
Maribellus comscasis                      0.585  0.0009 ***
Pusillimonas caeni                        0.584  0.0006 ***
Dongia mobilis                            0.583  0.0019 ** 
Desulfonatronum cooperativum              0.582  0.0008 ***
Desulfomicrobium hypogeium                0.582  0.0028 ** 
Anaerospora hongkongensis                 0.582  0.0015 ** 
Verticiella sediminum                     0.581  0.0010 ***
Sphingobacterium faecale                  0.579  0.0006 ***
Desulfovibrio porci                       0.578  0.0026 ** 
Veillonella infantium                     0.577  0.0017 ** 
Mucinivorans hirudinis                    0.576  0.0023 ** 
Fretibacterium fastidiosum                0.573  0.0020 ** 
```

Prolixibacter denitrificans	0.571	0.0016	**
Roseimarinus sediminis	0.571	0.0004	***
Horticoccus luteus	0.563	0.0059	**
Desulfoprunum benzoelyticum	0.557	0.0120	*
Lachnoclostridium pacaense	0.557	0.0137	*
Streptococcus varani	0.557	0.0125	*
Megasphaera indica	0.555	0.0036	**
Sphingomonas prati	0.554	0.0042	**
Eubacterium sulci ATCC 35585	0.547	0.0091	**
Thalassospira australica	0.546	0.0070	**
Cephaloticoccus capnophilus	0.545	0.0108	*
Geoalkalibacter ferrihydriticus	0.545	0.0108	*
Legionella hackeliae	0.545	0.0117	*
Elstera cyanobacteriorum	0.543	0.0020	**
Peptoclostridium acidaminophilum	0.542	0.0048	**
Veillonella magna	0.535	0.0046	**
Advenella mandrilli	0.535	0.0040	**
Azospirillum griseum	0.534	0.0125	*
Opitutus terrae PB90-1	0.534	0.0123	*
Sunxiuqinia rutila	0.534	0.0120	*
Alistipes montrealensis	0.533	0.0062	**
Pusillimonas soli	0.532	0.0006	***
Thiovirga sulfuroxydans	0.532	0.0001	***
Solidesulfovibrio carbinolicus	0.532	0.0058	**
Psychrosinus fermentans	0.531	0.0049	**
Aquimonas voraii	0.529	0.0070	**
Aminobacterium colombiense	0.527	0.0098	**
Lactivibrio alcoholicus	0.525	0.0047	**
Empedobacter falsenii genomovar 1	0.524	0.0065	**
Anaeroarcus burkinensis	0.524	0.0057	**
Holophaga foetida	0.524	0.0027	**
Halovulum marinum	0.522	0.0109	*
Millionella massiliensis	0.522	0.0125	*
Rhodopseudomonas palustris	0.522	0.0109	*
Legionella tunisiensis	0.520	0.0023	**
Acidaminococcus provencensis	0.519	0.0070	**
Kaistia geumhonensis	0.518	0.0032	**
Legionella qingyii	0.516	0.0015	**
Mangrovibacterium marinum	0.515	0.0075	**
Paracandidimonas soli	0.513	0.0041	**
Pusillimonas thiosulfatoxidans	0.512	0.0016	**
Pollutimonas nitritireducens	0.511	0.0043	**
Alistipes shahii WAL 8301	0.509	0.0083	**
Prosthecochloris marina	0.508	0.0050	**
Ancylobacter rudongensis	0.508	0.0092	**
Reyranella aquatilis	0.508	0.0056	**
Castellaniella defragrans	0.507	0.0083	**
Caenimicrobium hargitense	0.505	0.0026	**
Kaistia hirudinis	0.505	0.0095	**
Limisphaera ngatamarikiensis	0.504	0.0056	**
Pusillimonas ginsengisoli	0.502	0.0016	**
Caenibius tardaugens	0.502	0.0105	*
Dorea phocaeensis	0.502	0.0088	**
Parapusillimonas granuli	0.501	0.0035	**
Methylobacillus glycogenes	0.500	0.0101	*
Ignavibacterium album JCM 16511	0.499	0.0091	**
Legionella taurinensis	0.499	0.0074	**
Syntrophomonas bryantii	0.497	0.0142	*
Legionella pneumophila subsp. fraseri	0.497	0.0084	**
Tahibacter caeni	0.497	0.0062	**
Prolixibacter bellariivorans	0.496	0.0111	*
Desulfovibrio intestinalis	0.496	0.0069	**
Ampullimonas aquatilis	0.494	0.0060	**
Desulfuromonas svalbardensis	0.494	0.0202	*
Mangrovibacterium lignilyticum	0.494	0.0199	*
Castellaniella hirudinis	0.493	0.0123	*
Pusillimonas minor	0.493	0.0010	***
Achromobacter animicus	0.489	0.0105	*
Legionella norrlandica	0.489	0.0017	**
Labilibacter sediminis	0.488	0.0186	*
Aquabacter cavernae	0.488	0.0130	*
Desulfobulbus alkaliphilus	0.486	0.0135	*
Flavobacterium celericrescens	0.482	0.0062	**
Legionella santicrucis	0.480	0.0033	**
Flavobacterium cucumis	0.480	0.0177	*
Mariniphaga sediminis	0.480	0.0244	*
Solidesulfovibrio aerotolerans	0.473	0.0237	*

```
Veillonella atypica                         0.471  0.0150 *
Ralstonia solanacearum                      0.470  0.0203 *
Legionella drozanskii                       0.469  0.0153 *
Paracandidimonas caeni                      0.469  0.0033 **
Castellaniella denitrificans                0.469  0.0191 *
Oleisolibacter albus                        0.469  0.0107 *
Legionella saoudiensis                      0.468  0.0116 *
Magnetospira thiophila                      0.465  0.0430 *
Hydrogenoanaerobacterium saccharovorans     0.464  0.0166 *
Pirellula staleyi DSM 6068                  0.464  0.0156 *
Propionicimonas paludicola                  0.463  0.0204 *
Paludibacter propionicigenes WB4            0.461  0.0213 *
Achromobacter aloeverae                     0.457  0.0259 *
Bacteroides sedimenti                       0.457  0.0283 *
Eoetvoesia caeni                            0.457  0.0264 *
Legionella massiliensis                     0.456  0.0272 *
Azovibrio restrictus                        0.456  0.0204 *
Melioribacter roseus P3M-2                  0.455  0.0154 *
Lysobacter concretionis                     0.455  0.0259 *
Orrella dioscoreae                          0.453  0.0194 *
Oleomonas sagaranensis                      0.453  0.0284 *
Legionella rubrilucens                      0.451  0.0166 *
Achromobacter xylosoxidans                  0.451  0.0420 *
Thermanaeromonas burensis                   0.451  0.0250 *
Acetobacteroides hydrogenigenes             0.451  0.0269 *
Puteibacter caeruleilacunae                 0.447  0.0105 *
Bordetella avium                            0.446  0.0227 *
Bacteroides ihuae                           0.446  0.0295 *
Helcococcus sueciensis                      0.446  0.0326 *
Pusillimonas maritima                       0.445  0.0264 *
Rossellomorea arthrocnemi                   0.444  0.0498 *
Alkaliflexus imshenetskii                   0.442  0.0321 *
Alistipes putredinis                        0.440  0.0164 *
Bacteroides graminisolvens                  0.438  0.0147 *
Rothia endophytica                          0.434  0.0362 *
Pseudahrensia todarodis                     0.432  0.0433 *
Anaeromusa acidaminophila                   0.432  0.0426 *
Bifidobacterium adolescentis ATCC 15703     0.431  0.0375 *
Achromobacter denitrificans                 0.431  0.0384 *
Sporolituus thermophilus DSM 23256          0.429  0.0316 *
Bordetella petrii                           0.427  0.0398 *
Sphingosinicella xenopeptidilytica          0.427  0.0349 *
Oceanibaculum pacificum                     0.426  0.0346 *
Rhizobium tropici CIAT 899                  0.426  0.0396 *
Advenella faeciporci                        0.425  0.0240 *
Chryseobacterium reticulitermitis           0.425  0.0388 *
Azonexus hydrophilus DSM 23864              0.424  0.0259 *
Chelativorans alearense                     0.423  0.0487 *
Pelistega ratti                             0.422  0.0368 *
Paludisphaera soli                          0.422  0.0253 *
Hyphomonas polymorpha                       0.422  0.0377 *
Leucobacter salsicius M1-8                  0.421  0.0371 *
Bacteroides uniformis                       0.421  0.0406 *
Achromobacter insuavis                      0.418  0.0273 *
Carboxylicivirga mesophila                  0.417  0.0487 *
Candidimonas humi                           0.416  0.0464 *
Achromobacter dolens                        0.415  0.0443 *
Sporomusa acidovorans                       0.414  0.0484 *
Thalassospira lohafexi                      0.414  0.0444 *
Legionella rowbothamii                      0.413  0.0471 *
Laribacter hongkongensis                    0.411  0.0467 *
Hydromonas duriensis                        0.410  0.0495 *
Legionella sainthelensi                     0.408  0.0471 *
Solidesulfovibrio marrakechensis            0.408  0.0443 *
Pusillimonas noertemannii                   0.398  0.0474 *
Legionella longbeachae                      0.395  0.0131 *
Alysiella filiformis                        0.393  0.0354 *
Legionella waltersii                        0.374  0.0292 *
Aliarcobacter cibarius                      0.373  0.0496 *
Flavobacterium proteolyticum                0.361  0.0384 *
Legionella shakespearei                     0.342  0.0329 *

 Group PET  #sps.  39 
                                 stat p.value    
Acinetobacter beijerinckii      0.653  0.0010 ***
Edwardsiella anguillarum        0.583  0.0010 ***
[Clostridium] viride            0.532  0.0045 ** 
```

```
Propioniciclava soli             0.502  0.0143 *
Dysgonomonas mossii DSM 22836    0.495  0.0114 *
Ramlibacter alkalitolerans       0.490  0.0059 **
Aeromonas schubertii             0.480  0.0134 *
Acinetobacter defluvii           0.480  0.0085 **
Limnohabitans planktonicus II-D5 0.478  0.0158 *
Paraperlucidibaca baekdonensis   0.473  0.0214 *
Aeromonas taiwanensis            0.470  0.0189 *
Intestinimonas timonensis        0.470  0.0152 *
Endothiovibrio diazotrophicus    0.469  0.0283 *
Acinetobacter ihumii             0.454  0.0235 *
Citrobacter braakii              0.451  0.0248 *
Lawsonia intracellularis         0.450  0.0305 *
Lysobacter oligotrophicus        0.450  0.0328 *
Marinobacter oulmenensis         0.450  0.0311 *
Sphingomonas ginkgonis           0.450  0.0315 *
Chitinasiproducens palmae        0.449  0.0208 *
Sporobacter termitidis           0.447  0.0289 *
Peredibacter starrii             0.447  0.0316 *
Acinetobacter baretiae           0.444  0.0462 *
Aquabacterium limnoticum         0.440  0.0300 *
Pantoea dispersa                 0.440  0.0332 *
Tatumella punctata               0.440  0.0308 *
Sphingobium baderi LL03          0.439  0.0343 *
Acinetobacter qingfengensis      0.438  0.0233 *
Atlantibacter hermannii          0.432  0.0485 *
Inediibacterium massiliense      0.432  0.0303 *
Pseudenterobacter timonensis     0.424  0.0339 *
Giesbergeria sinuosa             0.423  0.0458 *
Parabacteroides chongii          0.421  0.0480 *
Clostridium sulfidigenes         0.420  0.0274 *
Citrobacter europaeus            0.420  0.0314 *
Acinetobacter plantarum          0.418  0.0478 *
Phreatobacter cathodiphilus      0.417  0.0500 *
Halothiobacillus neapolitanus    0.415  0.0469 *
Acinetobacter stercoris          0.370  0.0278 *

 Group PLA  #sps.  76
                                      stat p.value
Hydrogenophaga caeni                 0.697  0.0001 ***
Lysobacter chengduensis              0.690  0.0002 ***
Paracoccus siganidrum                0.676  0.0002 ***
Aromatoleum diolicum                 0.655  0.0017 **
Comamonas terrae                     0.641  0.0003 ***
Novosphingobium endophyticum         0.633  0.0023 **
Paracoccus sulfuroxidans             0.620  0.0003 ***
Sulfurifustis variabilis             0.617  0.0017 **
Oceaniglobus ichthyenteri            0.603  0.0014 **
Agitococcus lubricus                 0.587  0.0030 **
Paracoccus halophilus                0.576  0.0001 ***
Paracoccus mangrovi                  0.570  0.0023 **
Rhizobium giardinii                  0.568  0.0029 **
Gulbenkiania mobilis                 0.565  0.0038 **
Glutamicibacter protophormiae        0.557  0.0135 *
Ensifer adhaerens                    0.553  0.0033 **
Paracoccus lutimaris                 0.548  0.0039 **
Dechloromonas denitrificans          0.547  0.0035 **
Acidihalobacter prosperus            0.539  0.0078 **
Bartonella ancashensis               0.536  0.0119 *
Brucella papionis                    0.534  0.0112 *
Pseudogemmobacter bohemicus          0.532  0.0033 **
Erythrobacter dokdonensis            0.527  0.0084 **
Chelativorans multitrophicus         0.522  0.0119 *
Lysobacter oculi                     0.519  0.0019 **
Luteimonas yindakuii                 0.519  0.0076 **
Cypionkella sinensis                 0.513  0.0065 **
Xinfangfangia humi                   0.504  0.0034 **
Rhodobacter amnigenus                0.492  0.0078 **
Vitreoscilla stercoraria             0.492  0.0109 *
Brucella endophytica                 0.490  0.0145 *
Cypionkella collinsensis             0.486  0.0142 *
Paracoccus contaminans               0.483  0.0225 *
Paracoccus zhejiangensis             0.481  0.0161 *
Rhizobium halophytocola              0.481  0.0131 *
Xinfangfangia pollutisoli            0.479  0.0111 *
Pseudorhizobium tarimense            0.477  0.0160 *
Falsigemmobacter faecalis            0.475  0.0130 *
```

Paracoccus alimentarius	0.473	0.0163	*
Rhizobium indigoferae	0.472	0.0281	*
Abyssibacter profundi	0.469	0.0338	*
Hephaestia caeni	0.466	0.0188	*
Devosia oryziradicis	0.465	0.0443	*
Sphingobium jiangsuense	0.462	0.0104	*
Luteimonas terricola	0.462	0.0139	*
Ciceribacter azotifigens	0.462	0.0213	*
Croceibacterium xixiisoli	0.460	0.0254	*
Ciceribacter selenitireducens	0.458	0.0211	*
Pseudogemmobacter hezensis	0.458	0.0135	*
Empedobacter tilapiae	0.456	0.0166	*
Rhizobium arsenicireducens	0.452	0.0270	*
Eikenella corrodens	0.446	0.0375	*
Sphingopyxis ginsengisoli	0.446	0.0378	*
Diaphorobacter ruginosibacter	0.445	0.0261	*
Adhaeribacter terreus	0.444	0.0500	*
Rhizobium skierniewicense Ch11	0.444	0.0202	*
Pseudomonas wadenswilerensis	0.441	0.0357	*
Rhizobium rosettiformans W3	0.441	0.0269	*
Pseudomonas donghuensis	0.440	0.0314	*
Microbacterium saccharophilum	0.440	0.0384	*
Paracoccus koreensis	0.439	0.0305	*
Roseibium sediminis	0.436	0.0468	*
Mitsuaria chitinivorans	0.436	0.0372	*
Luteimonas notoginsengisoli	0.435	0.0472	*
Acinetobacter parvus	0.433	0.0392	*
Novosphingobium colocasiae	0.432	0.0442	*
Tsuneonella dongtanensis	0.432	0.0391	*
Dechlorobacter hydrogenophilus	0.431	0.0349	*
Novosphingobium chloroacetimidivorans	0.431	0.0444	*
Vogesella urethralis	0.426	0.0016	**
Paracoccus aminophilus	0.425	0.0415	*
Psychrobacter piechaudii	0.419	0.0452	*
Shinella fusca	0.417	0.0410	*
Mesorhizobium comanense	0.413	0.0486	*
Oryzomicrobium terrae	0.411	0.0500	*
Ensifer sesbaniae	0.400	0.0444	*

Group PVC.A #sps. 56

	stat	p.value	
Rhizobium pseudoryzae	0.785	0.0001	***
Caulobacter endophyticus	0.669	0.0001	***
Roseococcus pinisoli	0.668	0.0001	***
Limnohabitans australis	0.614	0.0012	**
Camelimonas fluminis	0.613	0.0009	***
Camelimonas lactis	0.606	0.0008	***
Desulfosporosinus fructosivorans	0.591	0.0009	***
Caulobacter segnis	0.581	0.0006	***
Chelatococcus reniformis	0.576	0.0025	**
Flaviflexus salsibiostraticola	0.543	0.0049	**
Acidovorax wautersii	0.542	0.0039	**
Caulobacter vibrioides	0.535	0.0052	**
Chelatococcus daeguensis	0.530	0.0053	**
Camelimonas abortus	0.527	0.0091	**
Arcanobacterium phocae	0.521	0.0086	**
Ancrocorticia populi	0.511	0.0071	**
Lysobacter xanthus	0.509	0.0100	**
Vagococcus hydrophili	0.506	0.0088	**
Youngiibacter fragilis 232.1	0.506	0.0132	*
Xanthomonas citri pv. malvacearum	0.506	0.0280	*
Comamonas thiooxydans	0.497	0.0073	**
Acidovorax cattleyae	0.494	0.0107	*
Pseudomonas mangrovi	0.489	0.0056	**
Comamonas testosteroni	0.484	0.0096	**
Pararhizobium mangrovi	0.483	0.0133	*
Pelosinus propionicus DSM 13327	0.483	0.0048	**
Methylobacterium durans	0.481	0.0149	*
Melaminivora jejuensis	0.477	0.0032	**
Microvirga subterranea	0.473	0.0175	*
Clostridium disporicum	0.468	0.0345	*
Methylovirgula ligni	0.466	0.0241	*
Acidovorax temperans	0.461	0.0193	*
Changpingibacter yushuensis	0.460	0.0130	*
Flaviflexus huanghaiensis	0.456	0.0105	*
Desulfitispora elongata	0.455	0.0259	*
Pseudomonas insulae	0.454	0.0183	*

Roseomonas cervicalis	0.448	0.0214	*
Merdimmobilis hominis	0.447	0.0338	*
Tessaracoccus coleopterorum	0.444	0.0274	*
Microvirga thermotolerans	0.443	0.0290	*
Caulobacter rhizosphaerae	0.438	0.0184	*
Simplicispira metamorpha	0.437	0.0358	*
Acidovorax antarcticus	0.432	0.0376	*
Anaerosinus glycerini	0.432	0.0084	**
Polaromonas cryoconiti	0.432	0.0343	*
Pseudomonas nitroreducens	0.429	0.0337	*
Acidovorax defluvii	0.429	0.0431	*
Phenylobacterium zucineum HLK1	0.428	0.0415	*
Microlunatus capsulatus	0.425	0.0452	*
Xenophilus arseniciresistens	0.424	0.0415	*
Massilibacillus massiliensis	0.423	0.0300	*
Rhodoferax koreense	0.419	0.0359	*
Acidovorax konjaci	0.417	0.0402	*
Clostridium polynesiense	0.417	0.0475	*
Acidovorax monticola	0.417	0.0463	*
Pseudomonas alcaligenes	0.393	0.0334	*

Group BR+PET #sps. 5

	stat	p.value	
Anaerosporomusa subterranea	0.493	0.0118	*
Tidjanibacter massiliensis	0.486	0.0123	*
Propionivibrio dicarboxylicus	0.469	0.0167	*
Microbacter margulisiae	0.441	0.0389	*
Limosilactobacillus mucosae	0.428	0.0385	*

Group BR+PLA #sps. 14

	stat	p.value	
Gemmobacter aquaticus	0.665	0.0002	***
Pseudostreptobacillus hongkongensis	0.540	0.0052	**
Alsobacter metallidurans	0.493	0.0134	*
Gemmobacter fontiphilus	0.489	0.0131	*
Novosphingobium bradum	0.481	0.0140	*
Azonexus caeni	0.470	0.0223	*
Novosphingobium aromaticivorans	0.455	0.0246	*
Novosphingobium arabidopsis	0.447	0.0304	*
Empedobacter brevis	0.446	0.0340	*
Gemmobacter aquatilis	0.430	0.0328	*
Xinfangfangia soli	0.427	0.0405	*
Novosphingobium subterraneum	0.416	0.0369	*
Euzebya pacifica	0.408	0.0431	*
Gemmobacter caeni	0.396	0.0391	*

Group BR+PVC.A #sps. 9

	stat	p.value	
Prosthecobacter dejongeii	0.501	0.0109	*
Prosthecobacter fluviatilis	0.500	0.0100	**
Lacibacterium aquatile	0.457	0.0153	*
Pseudobdellovibrio exovorus JSS	0.455	0.0254	*
Brevifollis gellanilyticus	0.448	0.0213	*
Phenylobacterium kunshanense	0.441	0.0238	*
Prosthecobacter algae	0.435	0.0293	*
Bdellovibrio bacteriovorus	0.420	0.0489	*
Tuwongella immobilis	0.418	0.0452	*

Group PET+PLA #sps. 86

	stat	p.value	
Acinetobacter calcoaceticus	0.603	0.0009	***
Aeromonas hydrophila	0.598	0.0013	**
Acinetobacter junii	0.591	0.0020	**
Acinetobacter radioresistens	0.588	0.0014	**
Comamonas denitrificans	0.570	0.0025	**
Acinetobacter lanii	0.565	0.0031	**
Acinetobacter kanungonis	0.564	0.0028	**
Acinetobacter shaoyimingii	0.563	0.0032	**
Acinetobacter wuhouensis	0.554	0.0036	**
Acinetobacter variabilis	0.551	0.0039	**
Acinetobacter refrigeratoris	0.550	0.0041	**
Acinetobacter tjernbergiae	0.550	0.0042	**
Acinetobacter rongchengensis	0.548	0.0046	**
Acinetobacter modestus	0.547	0.0044	**
Acinetobacter bouvetii	0.547	0.0037	**
Acinetobacter equi	0.545	0.0053	**
Acinetobacter albensis	0.543	0.0043	**

```
Acinetobacter baumannii                      0.542  0.0050 **
Comamonas aquatica subsp. rana               0.541  0.0046 **
Acinetobacter indicus                        0.541  0.0050 **
Acinetobacter courvalinii                    0.541  0.0051 **
Acinetobacter vivianii                       0.540  0.0052 **
Comamonas aquatilis                          0.539  0.0053 **
Acinetobacter tandoii                        0.537  0.0042 **
Acinetobacter haemolyticus                   0.531  0.0064 **
Acinetobacter johnsonii                      0.531  0.0056 **
Comamonas koreensis                          0.530  0.0049 **
Acinetobacter oryzae                         0.528  0.0064 **
Acinetobacter proteolyticus                  0.528  0.0059 **
Comamonas terrigena                          0.527  0.0068 **
Acinetobacter pittii DSM 21653               0.526  0.0050 **
Acinetobacter tianfuensis                    0.524  0.0070 **
Acinetobacter seohaensis                     0.524  0.0063 **
Comamonas jiangduensis                       0.524  0.0074 **
Allohahella antarctica                       0.521  0.0072 **
Comamonas kerstersii                         0.520  0.0059 **
Acinetobacter silvestris                     0.519  0.0071 **
Perlucidibaca piscinae                       0.515  0.0087 **
Pseudacidovorax intermedius                  0.513  0.0096 **
Acinetobacter baylyi                         0.513  0.0065 **
Aeromonas veronii bv. veronii                0.509  0.0094 **
Comamonas aquatica                           0.509  0.0086 **
Acinetobacter lactucae                       0.504  0.0108 *
Acinetobacter soli                           0.498  0.0128 *
Aeromonas lusitana                           0.497  0.0110 *
Mycoplana ramosa                             0.495  0.0088 **
Acinetobacter schindleri                     0.494  0.0117 *
Comamonas nitrativorans                      0.492  0.0149 *
Azonexus fungiphilus                         0.490  0.0143 *
Pseudaeromonas sharmana                      0.490  0.0139 *
Pseudorhodoferax soli                        0.489  0.0122 *
Paracoccus simplex                           0.487  0.0155 *
Acinetobacter bohemicus ANC 3994             0.482  0.0151 *
Cavicella subterranea                        0.482  0.0146 *
Acinetobacter bereziniae                     0.481  0.0149 *
Ciceribacter thiooxidans                     0.480  0.0141 *
Comamonas zonglianii                         0.477  0.0175 *
Acinetobacter oleivorans                     0.475  0.0173 *
Aquirhabdus parva                            0.470  0.0196 *
Acinetobacter halotolerans                   0.468  0.0178 *
Comamonas phosphati                          0.466  0.0219 *
Citrobacter murliniae                        0.465  0.0174 *
Acinetobacter lwoffii                        0.462  0.0227 *
Aeromonas veronii                            0.459  0.0254 *
Acinetobacter guillouiae                     0.456  0.0281 *
Acinetobacter seifertii                      0.456  0.0224 *
Acinetobacter indicus CIP 110367             0.456  0.0259 *
Acinetobacter towneri                        0.454  0.0268 *
Enterococcus aquimarinus                     0.453  0.0253 *
Serpentinimonas barnesii                     0.451  0.0274 *
Acinetobacter venetianus RAG-1 = CIP 110063 0.451  0.0305 *
Aeromonas dhakensis                          0.450  0.0267 *
Acinetobacter brisouii                       0.450  0.0251 *
Thalassomonas viridans                       0.445  0.0330 *
Pseudaeromonas pectinilytica                 0.445  0.0261 *
Rhizobium sullae                             0.444  0.0358 *
Rhizobium borbori                            0.444  0.0336 *
Acinetobacter apis                           0.443  0.0332 *
Acinetobacter nectaris CIP 110549            0.440  0.0354 *
Luteimonas granuli                           0.435  0.0342 *
Novosphingobium hassiacum                    0.434  0.0408 *
Pseudorhizobium marinum                      0.432  0.0360 *
Aeromonas enteropelogenes                    0.426  0.0457 *
Enterobacter wuhouensis                      0.424  0.0380 *
Pseudomonas argentinensis                    0.423  0.0440 *
Acinetobacter wanghuae                       0.416  0.0428 *

 Group PET+PVC.A  #sps.  2
                          stat p.value
Brevundimonas aurantiaca 0.473  0.0198 *
Brevundimonas viscosa    0.424  0.0380 *

 Group PLA+PVC.A  #sps.  1
                      stat p.value
```

```
Diaphorobacter caeni 0.498   0.008 **

 Group BR+PET+PLA  #sps.  4
                              stat p.value
Propionivibrio limicola      0.528  0.0053 **
Macellibacteroides fermentans 0.524  0.0059 **
Parabacteroides chartae      0.502  0.0095 **
Sphingomonas laterariae      0.417  0.0485 *

 Group PET+PLA+PVC.A  #sps.  2
                         stat p.value
Pseudomonas lalkuanensis 0.446  0.0286 *
Citrobacter gillenii     0.425  0.0409 *
---
Signif. codes:  0 ‘***’ 0.001 ‘**’ 0.01 ‘*’ 0.05 ‘.’ 0.1 ‘ ’ 1
```

Table S1: AMR genes detected using staramr tool

Gene	Predicted Phenotype	material	source
mph(E)	erythromycin, azithromycin	PLA	Inlet
msr(E)	erythromycin, azithromycin	PLA	Inlet
tet(39)	tetracycline	PLA	Inlet
sul1	sulfisoxazole	PET	Inlet
aph(6)-Id	kanamycin	PE	Inlet
sul2	sulfisoxazole	PE	Inlet
aac(3)-Ib	gentamicin	PLA	Outlet
blaBEL-1	ampicillin, amoxicillin/clavulanic acid, cefoxitin, ceftriaxone	PLA	Outlet
blaOXA-539	ampicillin	PLA	Outlet
qacE	unknown[qacE_1_X68232]	PLA	Outlet
sul1	sulfisoxazole	PLA	Outlet
blaOXA-58	ampicillin, meropenem	PET	Outlet
blaOXA-58	ampicillin, meropenem	PET	Outlet
sul1	sulfisoxazole	PE	Outlet

Table S2: Mobile genetic elements detected on chromosome using mobsuite tool.

molecule_type	contig_id	mge_type	mge_subtype	mge_length	material	source
chromosome	contig_5	ISPsy42	Tn3	5667	PLA	Inlet
chromosome	contig_5	ISPsy42	Tn3	5667	PLA	Inlet
chromosome	contig_365	IS6100	IS6	5466	PET	Inlet
chromosome	contig_365	IS6100	IS6	5466	PET	Inlet
chromosome	contig_75	IS401	IS3	1316	PET	Inlet
chromosome	contig_10	ISAlw1	IS5	1039	BR	Outlet
chromosome	contig_136	ISPpu12	ISL3	3372	BR	Outlet
chromosome	contig_262	ISAba12	IS5	1039	BR	Outlet
chromosome	contig_3	ISAba21	IS3	1274	BR	Outlet
chromosome	contig_544	ISAba14	IS3	1283	BR	Outlet
chromosome	contig_111	ISSod25	IS91	2313	PLA	Outlet
chromosome	contig_1144	ISAba125	IS30	2175	PLA	Outlet
chromosome	contig_121	ISPa22	IS1182	1669	PLA	Outlet
chromosome	contig_126	ISPa22	IS1182	1669	PLA	Outlet

chromosome	contig_129	ISCARN66	IS5	1200	PLA	Outlet
chromosome	contig_159	IS1474	IS21	2595	PLA	Outlet
chromosome	contig_161	ISPsme1	IS30	1066	PLA	Outlet
chromosome	contig_17	ISVapa4	IS21	2605	PLA	Outlet
chromosome	contig_190	ISPpu18	IS5	1192	PLA	Outlet
chromosome	contig_192	ISSpu5	IS21	2481	PLA	Outlet
chromosome	contig_20	ISAba21	IS3	1274	PLA	Outlet
chromosome	contig_223	ISPa1635	IS4	1637	PLA	Outlet
chromosome	contig_223	ISButh6	IS5	1331	PLA	Outlet
chromosome	contig_223	ISButh6	IS5	1331	PLA	Outlet
chromosome	contig_224	ISPpu18	IS5	1192	PLA	Outlet
chromosome	contig_225	ISCARN66	IS5	1200	PLA	Outlet
chromosome	contig_269	ISPpu18	IS5	1192	PLA	Outlet
chromosome	contig_349	ISSod25	IS91	2313	PLA	Outlet
chromosome	contig_387	IS1474	IS21	2595	PLA	Outlet
chromosome	contig_4	ISPa1635	IS4	1637	PLA	Outlet
chromosome	contig_4	ISRme10	IS30	1113	PLA	Outlet
chromosome	contig_404	ISApr11	IS1380	1671	PLA	Outlet
chromosome	contig_405	ISApr11	IS1380	1671	PLA	Outlet
chromosome	contig_48	ISRme10	IS30	1113	PLA	Outlet
chromosome	contig_523	ISRme10	IS30	1113	PLA	Outlet
chromosome	contig_61	ISAba125	IS30	2175	PLA	Outlet
chromosome	contig_64	IS1474	IS21	2595	PLA	Outlet
chromosome	contig_735	ISAba12	IS5	1039	PLA	Outlet
chromosome	contig_75	ISPpu12	ISL3	3372	PLA	Outlet
chromosome	contig_75	ISVapa4	IS21	2605	PLA	Outlet
chromosome	contig_78	ISButh6	IS5	1331	PLA	Outlet
chromosome	contig_78	ISPpu12	ISL3	3372	PLA	Outlet
chromosome	contig_81	ISRme10	IS30	1113	PLA	Outlet
chromosome	contig_82	IS1474	IS21	2595	PLA	Outlet
chromosome	contig_82	ISSpu5	IS21	2481	PLA	Outlet
chromosome	contig_844	ISStma11	ISL3	4426	PLA	Outlet
chromosome	contig_85	ISVapa4	IS21	2605	PLA	Outlet
chromosome	contig_879	ISSba6	IS4	1420	PLA	Outlet
chromosome	contig_883	ISCARN66	IS5	1200	PLA	Outlet
chromosome	contig_92	ISRme10	IS30	1113	PLA	Outlet
chromosome	contig_92	ISCARN66	IS5	1200	PLA	Outlet
chromosome	contig_1283	ISAba12	IS5	1039	PET	Outlet
chromosome	contig_1290	ISPpu18	IS5	1192	PET	Outlet
chromosome	contig_1458	ISRme10	IS30	1113	PET	Outlet
chromosome	contig_1505	ISPa22	IS1182	1669	PET	Outlet
chromosome	contig_165	ISAba125	IS30	2175	PET	Outlet
chromosome	contig_167	ISAba125	IS30	2175	PET	Outlet
chromosome	contig_168	ISAba12	IS5	1039	PET	Outlet
chromosome	contig_168	ISAba125	IS30	2175	PET	Outlet
chromosome	contig_173	ISAlw1	IS5	1039	PET	Outlet

chromosome	contig_174	ISAba17	IS66	2491	PET	Outlet
chromosome	contig_175	ISAba14	IS3	1283	PET	Outlet
chromosome	contig_175	ISAba125	IS30	2175	PET	Outlet
chromosome	contig_183	ISAba21	IS3	1274	PET	Outlet
chromosome	contig_183	ISAba21	IS3	1274	PET	Outlet
chromosome	contig_183	ISAba21	IS3	1274	PET	Outlet
chromosome	contig_19	ISAba11	IS701	1101	PET	Outlet
chromosome	contig_19	ISAba14	IS3	1283	PET	Outlet
chromosome	contig_191	ISSpe2	IS110	1366	PET	Outlet
chromosome	contig_212	ISAba12	IS5	1039	PET	Outlet
chromosome	contig_226	IS1474	IS21	2595	PET	Outlet
chromosome	contig_227	ISRme10	IS30	1113	PET	Outlet
chromosome	contig_271	ISSpe2	IS110	1366	PET	Outlet
chromosome	contig_286	ISSpe2	IS110	1366	PET	Outlet
chromosome	contig_300	ISStma11	ISL3	4426	PET	Outlet
chromosome	contig_300	ISStma11	ISL3	4426	PET	Outlet
chromosome	contig_350	ISSde6	IS3	1235	PET	Outlet
chromosome	contig_350	ISSod25	IS91	2313	PET	Outlet
chromosome	contig_37	ISRme10	IS30	1113	PET	Outlet
chromosome	contig_37	ISPpu12	ISL3	3372	PET	Outlet
chromosome	contig_37	ISButh6	IS5	1331	PET	Outlet
chromosome	contig_37	ISPst2	ISL3	2985	PET	Outlet
chromosome	contig_37	ISButh6	IS5	1331	PET	Outlet
chromosome	contig_40	ISAba12	IS5	1039	PET	Outlet
chromosome	contig_40	ISAlw1	IS5	1039	PET	Outlet
chromosome	contig_406	ISAba125	IS30	2175	PET	Outlet
chromosome	contig_406	ISAba125	IS30	2175	PET	Outlet
chromosome	contig_414	ISSba6	IS4	1420	PET	Outlet
chromosome	contig_457	ISAba17	IS66	2491	PET	Outlet
chromosome	contig_498	ISAeme12	IS4	1426	PET	Outlet
chromosome	contig_498	ISKpn26	IS5	1197	PET	Outlet
chromosome	contig_56	ISAs31	IS3	1322	PET	Outlet
chromosome	contig_6	ISAba24	IS66	2421	PET	Outlet
chromosome	contig_695	ISApr11	IS1380	1671	PET	Outlet
chromosome	contig_856	ISSde6	IS3	1235	PET	Outlet
chromosome	contig_856	ISSde6	IS3	1235	PET	Outlet
chromosome	contig_90	ISPa1635	IS4	1637	PET	Outlet
chromosome	contig_906	ISKpn31	ISAs1	1441	PET	Outlet
chromosome	contig_925	ISAba21	IS3	1274	PET	Outlet
chromosome	contig_96	ISRme10	IS30	1113	PET	Outlet
chromosome	contig_96	ISPst3	IS21	2606	PET	Outlet
chromosome	contig_96	ISPst3	IS21	2606	PET	Outlet
chromosome	contig_962	ISCARN66	IS5	1200	PET	Outlet
chromosome	contig_103	ISKpn26	IS5	1197	PE	Outlet
chromosome	contig_138	ISSpe2	IS110	1366	PE	Outlet
chromosome	contig_172	ISVapa4	IS21	2605	PE	Outlet

chromosome	contig_172	ISPa22	IS1182	1669	PE	Outlet
chromosome	contig_173	IS1474	IS21	2595	PE	Outlet
chromosome	contig_203	ISAs17	IS3	1333	PE	Outlet
chromosome	contig_282	ISApr11	IS1380	1671	PE	Outlet
chromosome	contig_317	ISPpu18	IS5	1192	PE	Outlet
chromosome	contig_324	ISRme10	IS30	1113	PE	Outlet
chromosome	contig_425	ISPpu12	ISL3	3372	PE	Outlet
chromosome	contig_44	ISSpe2	IS110	1366	PE	Outlet
chromosome	contig_466	ISSde6	IS3	1235	PE	Outlet
chromosome	contig_474	ISVapa4	IS21	2605	PE	Outlet
chromosome	contig_59	ISSba6	IS4	1420	PE	Outlet
chromosome	contig_601	ISPpu18	IS5	1192	PE	Outlet
chromosome	contig_677	ISAs31	IS3	1322	PE	Outlet

Table S3: Mobile genetic elements detected on plasmid using mobsuite tool

**molecule_type**	**contig_id**	**mge_type**	**mge_subtype**	**mge_length**	**material**	**source**
plasmid	contig_1470	ISPa38	Tn3	3400	PET	Outlet
plasmid	contig_573	ISAba11	IS701	1101	PET	Outlet
